# *Drosophila melanogaster* as a Model to Study the Multiple Phenotypes, Related to Genome Stability of the Fragile-X Syndrome

**DOI:** 10.3389/fgene.2019.00010

**Published:** 2019-02-13

**Authors:** Valeria Specchia, Antonietta Puricella, Simona D’Attis, Serafina Massari, Angela Giangrande, Maria Pia Bozzetti

**Affiliations:** ^1^ Dipartimento di Scienze e Tecnologie Biologiche ed Ambientali, DiSTeBA, Università del Salento, Lecce, Italy; ^2^ Institut de Génétique et de Biologie Moléculaire et Cellulaire, Illkirch, France; ^3^ Centre National de la Recherche Scientifique, UMR7104, Illkirch, France; ^4^ Institut National de la Santé et de la Recherche Médicale, U964, Illkirch, France; ^5^ Université de Strasbourg, Illkirch, France

**Keywords:** FMRP/dFmr1, Fragile-X syndrome, piRNA pathway, DNA damage response, transposon elements, neurological diseases

## Abstract

Fragile-X syndrome is one of the most common forms of inherited mental retardation and autistic behaviors. The reduction/absence of the functional FMRP protein, coded by the X-linked *Fmr1* gene in humans, is responsible for the syndrome. Patients exhibit a variety of symptoms predominantly linked to the function of FMRP protein in the nervous system like autistic behavior and mild-to-severe intellectual disability. Fragile-X (FraX) individuals also display cellular and morphological traits including branched dendritic spines, large ears, and macroorchidism. The *dFmr1* gene is the Drosophila ortholog of the human *Fmr1* gene. *dFmr1* mutant flies exhibit synaptic abnormalities, behavioral defects as well as an altered germline development, resembling the phenotypes observed in FraX patients. Therefore, *Drosophila melanogaster* is considered a good model to study the physiopathological mechanisms underlying the Fragile-X syndrome. In this review, we explore how the multifaceted roles of the FMRP protein have been addressed in the *Drosophila* model and how the gained knowledge may open novel perspectives for understanding the molecular defects causing the disease and for identifying novel therapeutical targets.

## Introduction

**Figure 1:**
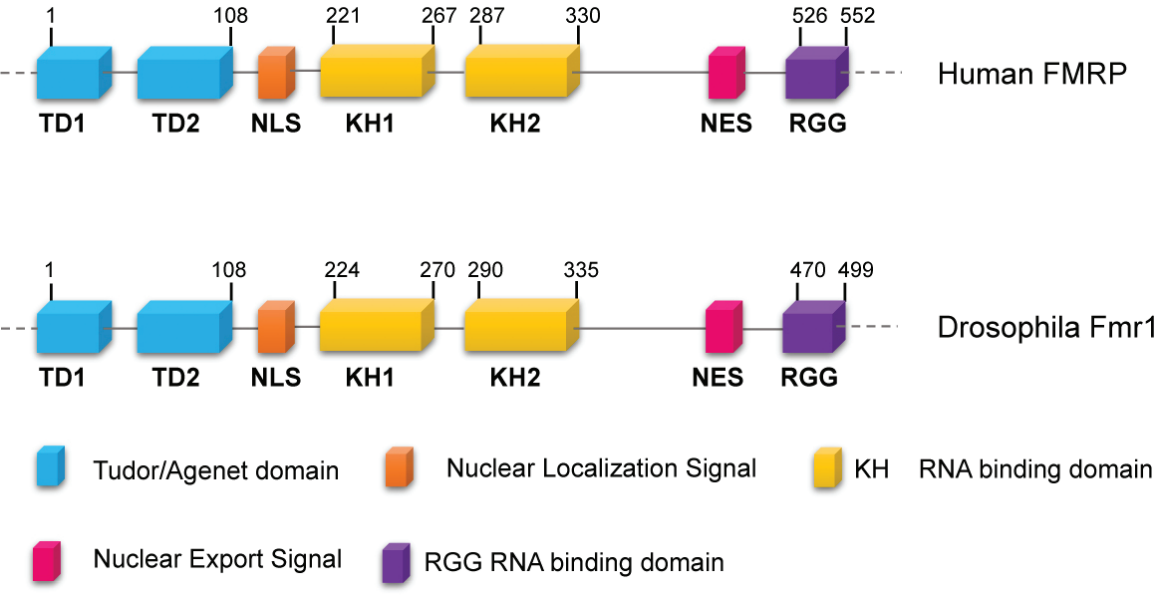
Conserved domains of FMRP/dFmr1 proteins. The drawings are not to scale; the exact positions of the amino acids are indicated; the domains are indicated with different colors.

Fragile-X syndrome (FXS, MIM300624) is the most common form of mental retardation in the human population. This affects approximately 1/7,000 males and 1/11,000 females (Hunter et al., 2014), and patients exhibit intellectual disability, autism, hyperactivity, long face, large ears, language delay, hyper arousal anxiety (Johannisson et al., 1987; O’Donnell and Warren, 2002; Santoro et al., 2012) macroorchidism, and malformed spermatids (Johannisson et al., 1987; Slegtenhorst-Eegdeman et al., 1998). The most frequent cause of the syndrome is a CGG trinucleotide repeat expansion (greater than 200 repeats) in the 5′ of the Fragile-X locus in Xq27.3, which leads to the hypermethylation of the gene promoter. The final effect is the transcriptional silencing of the Fragile-X Mental Retardation (*Fmr1*) gene, with a consequent loss of the encoded FMRP protein (Godler et al., 2010). FMRP is a complex protein that displays distinct motifs: a nuclear localization signal (NLS) and a nuclear export signal (NES), two tandem Tudor domains that are likely involved in protein-protein interactions and/or in the DNA binding, as well as three RNA-binding domains including two KH domains and one Arg-Gly-Gly (RGG) box (Figure 1) (O’Donnell and Warren, 2002; Ramos et al., 2006; Santoro et al., 2012). In mammals, FMRP is nearly ubiquitous, but it is heavily expressed in neurons, particularly in the cortex, hippocampus, and Purkinje cells where it regulates specific messenger targets. FMRP is also expressed at high levels in testes. Accordingly, the main effects of the FMRP loss in humans are in the nervous system and in the gonads (Santoro et al., 2012). In neurons, the absence of FMRP may alter the processing, the localization, and/or the translational regulation of mRNAs encoding pre- and postsynaptic proteins. These defects can account for the abnormal maturation of dendritic spines in FXS patients, which are longer, thinner, and denser than the normal ones (Swanger and Bassell, 2011; Bardoni et al., 2012; Maurin et al., 2014), representing the cellular defects underpinning the neuronal dysfunctions characterizing the Fragile-X disorder.

In addition to CGG triplet expansion, different mutations in the *Fmr1* gene, leading to FXS, have been reported. They include deletions and missense and nonsense mutations, which are listed in the Human Gene Mutation Database for FXS^1^. Mutations occur all along the coding sequences and affect different domains, which may explain why the FraX patients display common as well as specific defects (Reeve et al., 2008; Santoro et al., 2012; Alpatov et al., 2014; Okray et al., 2015; Suhl and Warren, 2015; Quartier et al., 2017).

Two autosomal homologs of *Fmr1* have been identified in the human genome: the Fragile-X mental retardation autosomal homolog 1 (FXR1) and 2 (FXR2), together with the *Fmr1* gene, form the Fragile-X gene family (Siomi et al., 1995; Zhang et al., 1995). Both homologs encode for RNA-binding proteins, FXR1P and FXR2P, with similar and/or complementary functions to those of FMRP, respectively (Penagarikano et al., 2007; Ascano et al., 2012).

A particular aspect linked to FXS is that individuals with a number of CGG repeats from 55 to 200 present a condition known as premutation and display an increased amount of *Fmr1* mRNA. It was proposed that the symptoms, exhibited by these subjects, are related to the *Fmr1* mRNA overproduction. Males with the premutation are at risk to developing Fragile-X-associated tremor/ataxia syndrome (FXTAS, MIM300623), whereas females with the premutation have an increased probability to develop Fragile-X-associated primary ovary insufficiency (FXPOI) (Amiri et al., 2008; Kronquist et al., 2008; Rossetti et al., 2017).

The function of FMRP has been primarily studied in the nervous system of mammals and *Drosophila*, focusing on its role as a translational regulator acting: either by repressing translational initiation (Schenck et al., 2003; Napoli et al., 2008; Aitken and Lorsch, 2012) or by interacting with the translating ribosomes (Siomi et al., 1996; Tamanini et al., 1996; Feng et al., 1997; Ishizuka et al., 2002; Darnell et al., 2005). It has also been proposed that FMRP may exert its translational control through the miRNA pathway (Siomi et al., 1996; Caudy et al., 2002; Ishizuka et al., 2002; Jin et al., 2004; Xu et al., 2008). Many screenings, aiming at identifying FMRP targets (mRNAs and proteins), contributed to the understanding of the role of FMRP, mainly in the nervous system. Many of these targets are involved in synaptic activity, which may account for the FXS phenotypes, such as defects in the development of neuronal architecture and in synaptic dysfunction (Darnell et al., 2011; Ascano et al., 2012).

FMRP regulates the local translation of a subset of mRNAs at synapses following the activation of the metabotropic glutamate receptors (mGluRs) (Huber et al., 2002; Bear et al., 2004; McBride et al., 2005). Deregulation of local protein synthesis is considered a core mechanism in FXS, underlying altered synaptic plasticity and consequent cognitive impairment. The role of FMRP in the regulation of translation was better characterized in the Drosophila quiescent oocyte in which the translation of stored mRNAs is a crucial point for the correct development of embryos (Greenblatt and Spradling, 2018).

Animal models of FXS have been developed in zebrafish, mouse, and rat (Tucker et al., 2004; McBride et al., 2005, 2012; Hamilton et al., 2014). Over the last decades, *Drosophila* has also provided key contributions to further understand the molecular pathways defective in FXS, thanks to the many advantages in the use of this versatile organism (Tessier and Broadie, 2012; Sears and Broadie, 2017; Drozd et al., 2018; Dockendorff and Labrador, 2019). The resulting imprecise excisions provided *Fmr1* alleles that lack dFmr1 expression, a situation comparable to the loss of function mutations observed in FXS patients (Wan et al., 2000). dFmr1 is equally similar to the three mammalian gene products (~35% identity, ~60% similarity) and shows particularly high sequence conservation (~70% identity) in critical domains such as the Tudor/Agenet domain that is involved in DNA binding, the RNA-binding domains, and the nuclear localization signals (Zalfa et al., 2007; Zhang et al., 2007; Xu et al., 2008).

**Figure 2:**
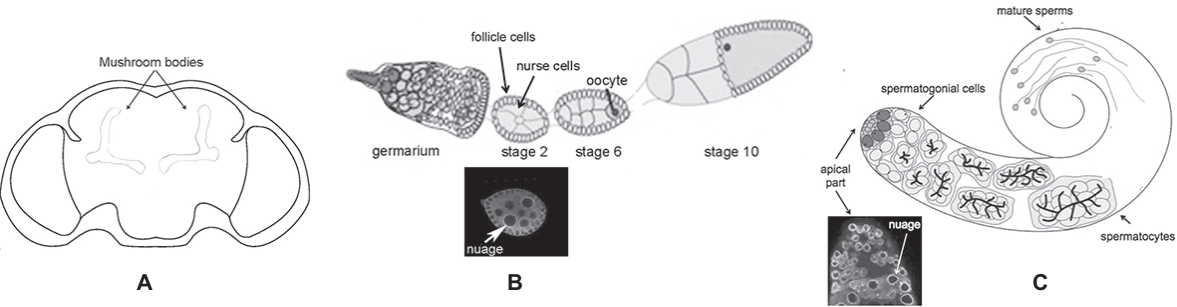
Schematic of different body parts of a *Drosophila melanogaster* adult. **(A)** Head, the mushroom bodies are indicated. **(B)** Upper part: ovariole; lower part: immunolabeling of a stage 2 oocyte; the white arrow indicates the perinuclear nuage. **(C)** Upper part: adult testis; lower part: immunolabeling of the apical part of the testis is indicated; the white arrow indicates the perinuclear nuage.

The *Drosophila melanogaster* dFmr1 protein is expressed from embryonic stages to adult, and it is enriched in the nervous system (Morales et al., 2002). In the brain, dFmr1 is highly expressed in the mushroom bodies, the main structure of the brain involved in cognitive functions. dFmr1 highly accumulates in the dendrites and in the axons of Kenyon cells, the intrinsic neurons of the mushroom bodies (Figure 2A). Its expression is ubiquitous in the neurons of the adult brain, whereas very low levels have been detected in glial cells (Wan et al., 2000; Zhang et al., 2001; Morales et al., 2002; Coffee et al., 2010). Outside the nervous system, dFmr1 is presented at a high level in larval and adult testes with a strong expression in spermatocytes (Zhang et al., 2004; Bozzetti et al., 2015). dFmr1 is also a component of the polar granules of the embryo where it interacts with other specific proteins present in these structures such as Vasa, Cup, and Hsp83 (Verrotti and Wharton, 2000; Cziko et al., 2009; Pisa et al., 2009; Lasko, 2013).

The *Drosophila* animals that completely lack dFmr1 recapitulate many of the phenotypes exhibited by patients with the Fragile-X syndrome. At the cellular level, mutants present defective neuronal architecture and synaptic function. The neurons of *dFmr1* null mutant animals exhibit abnormally organized synapses in both the peripheral and central nervous systems. The neuromuscular junctions (NMJs) of the *Drosophila* larva are simple synapses that represent a good model to study synaptic plasticity. The lack of *dFmr1* causes pronounced synaptic overgrowth at the NMJs (Zhang et al., 2001; Schenck et al., 2003; Pan et al., 2004). Mutant flies display altered behaviors, such as reduced courtship activity of males and irregular circadian rhythms, like the eclosion timing, even though the mRNAs for the two clock elements Per and Tim are not affected. In addition, *dFmr1* mutants exhibit defects in locomotor activity and an acute impairment of long-term memory (Sehgal et al., 1994; Dockendorff et al., 2002; Morales et al., 2002; Bolduc et al., 2008, 2010).

In the ovary, dFmr1 plays a role in translational regulation (Costa et al., 2005), where it controls germ stem cell differentiation through the miRNA-mediated pathway (Yang et al., 2007) and cell proliferation through the proto-oncogene *cbl* (Epstein et al., 2009).

Interestingly, dFmr1 is also involved in the piRNA pathway in the *Drosophila* gonads as well as in the DNA damage response in *Drosophila* and mouse (Zhang et al., 2012; Alpatov et al., 2014; Bozzetti et al., 2015) These findings provide a direct link between dFmr1/FMRP (from here onward, we will name dFmr1 the Drosophila protein as FMRP the mammalian protein) and genome instability, which may represent the common denominator for the multiple phenotypes described in the Fragile-X syndrome and in animal models for the disease.

In this review, we will predominantly treat the roles of dFmr1 related to the genome instability in the gonads and in the nervous system.

## The Role of dFmr1 in the piRNA Pathway

### *dFmr1* Mutations Affect the Regulation of the *Crystal-Stellate* System and of the Transposable Elements in the Gonads

In 2015, our group demonstrated, for the first time, the role of dFmr1 in the piRNA-mediated silencing of transposable elements and repetitive sequences in the *Drosophila* gonads (Bozzetti et al., 2015). Piwi-interacting RNAs or piRNAs are small RNA molecules protecting animal germ cells and their somatic precursors from the insertion of transposons and other repetitive elements hence preserving genome stability (Malone et al., 2009; Patil and Kai, 2010; Zhang et al., 2011; Anand and Kai, 2012; Specchia et al., 2017). The genomic clusters that act as sources of piRNAs contain multiple and also defective transposon sequences. Most of the piRNA clusters produce piRNAs from both genomic strands, and the other clusters produce piRNAs only from one genomic strand.

The molecular mechanism underlying the silencing of transposable elements reached a deep level of knowledge following studies performed in the ovaries. Argonaute proteins, belonging to the Piwi subfamily groups (P-element-induced Wimpy Testes or Piwi, Aubergine or Aub, and Ago3), play a crucial role in these processes (Aravin et al., 2007). Aub and Ago3 localize to the nuage (Figure 2B), a perinuclear structure found in animal germ cells. Piwi localizes predominantly in the nucleus of both germ and somatic cells of the ovary.

**Figure 3:**
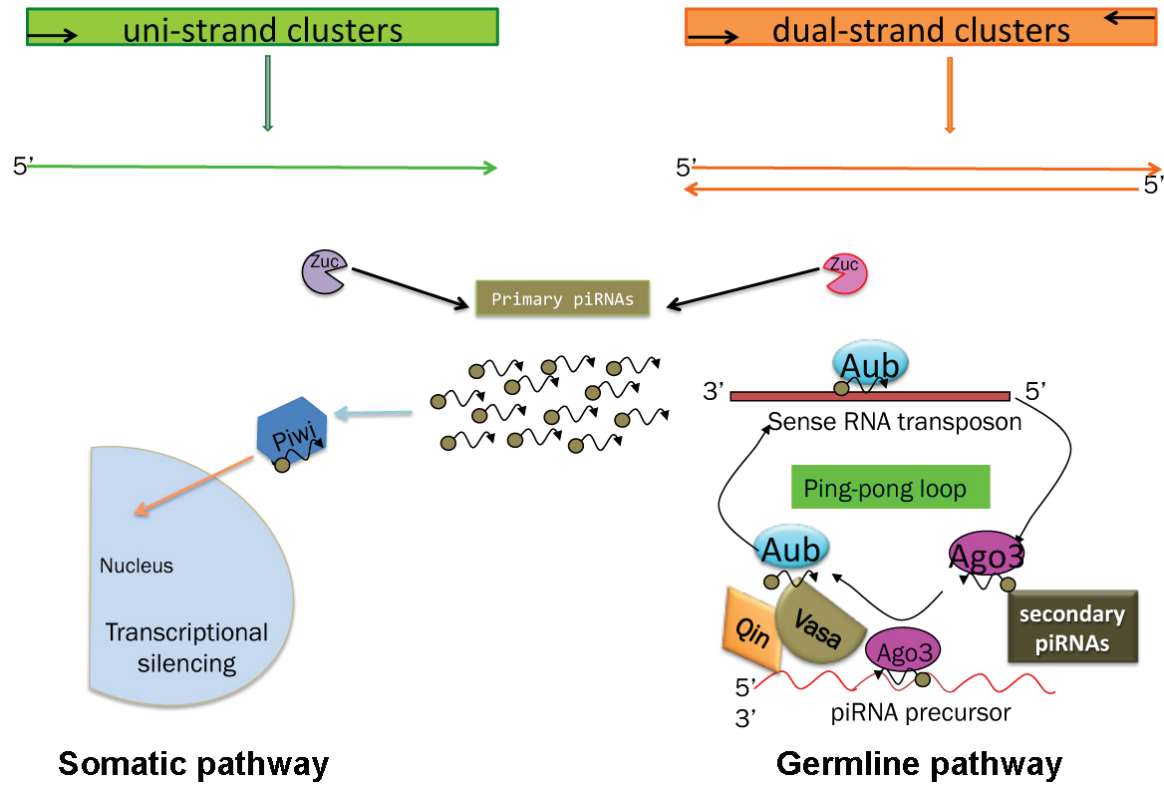
Schematic of the piRNA’s biogenesis. Somatic and germline pathways are indicated. Zuc stands for Zucchini protein (see text). In the germline pathway, Qin is a partner of Vasa, which behaves like a molecular platform for the piRNA pathway (see text and Specchia et al., 2017).

Two pathways for piRNA biogenesis and function have been established the primary and the ping-pong pathways (Figure 3) (Aravin et al., 2007; Malone et al., 2009).

In the primary pathway, transcript precursors, arising from specific genomic clusters, are processed into primary piRNAs that are bound by specific Piwi proteins. *Drosophila* ovarian somatic cells use exclusively the primary pathway. In these cells, the process occurs in perinuclear Yb bodies, which are discrete cytoplasmic compartments that take their name from the principal player in the process, the protein Yb, in the somatic cells of the ovary and testis (Szakmary et al., 2009). piRNA factors, such as Armitage, Shutdown, and Vreteno, accumulate in the Yb bodies. Upon the formation of the 3′ end of the precursors by Zucchini, the mature primary piRNAs are loaded onto Piwi, which then enters the nucleus and induces transcriptional transposon silencing (Saito et al., 2010).

Germ cells use predominantly the ping-pong amplification process in which the primary piRNAs are subjected to an amplification loop that increases their amount. In this case, the Piwi subfamily proteins, Aub and Ago3, bind the piRNAs and use the sequence homology to recognize the corresponding transposon transcript. Aub and Ago3 cooperate in the ping-pong pathway to amplify the piRNAs (Aravin et al., 2007).

The primary and the ping-pong pathways are also present in *Drosophila* testes (Figure 2C). In this tissue, the most abundant piRNAs associated with Aub and Ago3 correspond to the “*crystal*” piRNAs (Aravin et al., 2001, 2003; Vagin et al., 2006; Nishida et al., 2007; Bozzetti et al., 2012). The *crystal-Stellate* system represents the first reported natural case of piRNA-mediated regulation, where the repetitive euchromatic *Stellate* sequences are silenced by the piRNAs produced by the heterochromatic *crystal* locus. *Stellate* and *crystal* are composed of tens to hundreds of copies of repetitive sequences organized in tandem (Livak, 1984; Palumbo et al., 1994; Belloni et al., 2002; Tritto et al., 2003; Egorova et al., 2009; Bozzetti et al., 2012). At the molecular level, the loss of the *crystal* region or the “loss of function” mutations of genes involved in the *crystal-Stellate* regulation, called *crystal-Stellate* modifiers, results in the production of a testes-specific *Stellate* mRNA of 750 bases, coding for the Stellate protein. This results in the formation of needle or star-shaped crystalline aggregates that can be revealed by using a specific antibody (Bozzetti et al., 1995). The phenotype induced by *crystal-Stellate* misregulation has provided an efficient tool to identify several genes involved in the piRNA pathway. The majority of the *crystal-Stellate* modifiers has a role in the silencing of germinal and somatic transposons and participates in the primary as well as in the ping-pong pathway. Interestingly, mutants for these genes affect fertility, at various degrees, both in females and males (Pane et al., 2007; Specchia et al., 2008, 2017; Specchia and Bozzetti, 2009; Bozzetti et al., 2012; Sahin et al., 2016).

Null *dFmr1* mutations affect the piRNA pathway in the gonads and the fertility of males and females (Zhang et al., 2004; Bozzetti et al., 2015). In the mutant testes, the levels of the “*crystal*” specific piRNAs are reduced, leading to the formation of the crystalline aggregates. In addition, dFmr1 was demonstrated to have a role in the piRNA-mediated silencing of both germline and somatic transposable elements (TEs) (Bozzetti et al., 2015). For all these reasons, dFmr1 should be considered as a *bona fide* component of the piRNA pathway, at least in the gonads. More recently, the role in the silencing of TEs was confirmed by the work of Jiang et al. who demonstrated that the expression of selfish genetic elements increases in the ovaries of *dFmr1* mutant females (Jiang et al., 2016).

### dFmr1 Genetic and Biochemical Interaction With Argonaute Proteins in the Gonads and in the Nervous System

The Argonaute proteins are key players of the small RNA-mediated silencing pathway, being the components of the RNA-induced silencing complex (RISC). By using small RNA molecules, they mediate the post-transcriptional control of repetitive sequences, transposons, and genes in different tissues (Kalmykova et al., 2005; Brennecke et al., 2007; Klattenhoff and Theurkauf, 2008; Zhou et al., 2008; Li et al., 2009; Malone et al., 2009). The *Drosophila melanogaster* genome contains five genes coding for proteins of Argonaute family: Ago1 and Ago2 belong to the Ago subfamily and work in the miRNA (micro RNA) and siRNA (small interfering RNA) pathways. As mentioned above, Ago3, Piwi, and Aub act predominantly in the gonad-specific piRNA pathway (Li et al., 2009; Thomson and Lin, 2009).

Ago1 is commonly associated to the miRNA pathway, but data from our lab assign to this protein an additional role in the piRNA pathway as well. Indeed, Ago1 affects the silencing of the transposons in the gonads of both sexes, is involved in *crystal-Stellate* regulation in the *Drosophila* testis (Bozzetti et al., 2015; Specchia et al., 2017), and localizes at the “nuage” in the subcellular compartment in which other piRNA components localize, at least in testes (Kibanov et al., 2011; Nagao et al., 2011). Accordingly, an Ago1-mediated function was demonstrated to be required for the formation of piRNAs in follicle cells, linking together the two pathways (Mugat et al., 2015). The Ago1 protein, hence, has a promiscuous role in small RNA regulation.

A strong argument supporting the role of dFmr1 in the small RNA-mediated pathways is the finding that dFmr1 interacts with the Argonaute proteins. One of the first evidence was provided by the biochemical interaction of dFmr1 with Ago2 and with the components of the RISC in S2 *Drosophila* cells (Caudy et al., 2002; Ishizuka et al., 2002).

Since this discovery, many efforts were made to clarify the molecular role of FMRP in the RNA-mediated silencing pathways based on the genetic and biochemical interactions with the Argonaute proteins. Almost all the Argonaute proteins of both subfamilies have been connected to dFmr1 in the gonads as well as in the nervous system. We here present the main findings related to the specific role of FMRP in the small RNA pathways in the two tissues, disclosing multifaceted connections.

dFmr1 interacts with Ago1 and with the *bantam* microRNA in the *Drosophila* ovary to regulate the fate of germline stem cells (Yang et al., 2007, 2009). Ago1 was also implicated in terminal dendrites elongation (Lee et al., 2015) and is required for a correct function of dFmr1 at the NMJ (Jin et al., 2004; Bozzetti et al., 2015).

**Figure 4:**
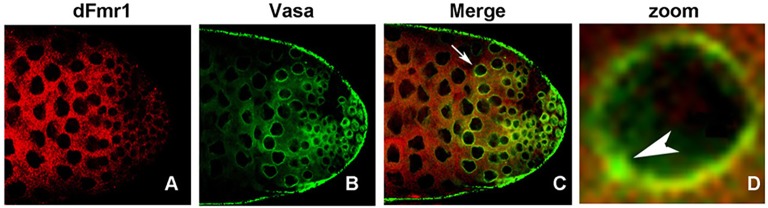
dFmr1 and Vasa immunolocalization in wt (wild type) adult testes. **(A)** Single confocal section of a wt testis labeled with anti-dFmr1, **(B)** anti-Vasa, and **(C)** merge; magnification 40×. **(D)** Photographic zoom of the cells indicated by arrow in **(C)**; the white arrowhead shows the colocalization of dFmr1 and Vasa in the piNG body.

dFmr1 also interacts genetically with Aub, whose overexpression in the germline, as well as in the somatic tissues of the *dFmr1* mutant animals, rescues the phenotypes related to the regulation of transposable elements and to the *crystal-Stellate* interaction mediated by piRNAs (Bozzetti et al., 2015). dFmr1 is widely distributed in the gonads, and it overlaps with Aub at the nuage and at the “piRNA nuage giant bodies” (piNG bodies) (Figure 4), a giant structure in the nuage of testes where the piRNA components are located and function (Bozzetti et al., 2015). The biochemical interaction between dFmr1 and Aub, in S2 cells, also supports the data obtained with the genetic experiment (Bozzetti et al., 2015). Aub and dFmr1 were demonstrated also to genetically interact in the larval neuromuscular junctions, as the neuronal overexpression of *aub* rescues the *dFmr1* defective NMJs (Bozzetti et al., 2015). Since the presence of Aub in the nervous system is still debated (see the following paragraphs), it has been proposed that the overexpressed Aub may work by taking on the function of Ago1, a protein that is definitely present and has a well-studied role in the nervous system (Lee et al., 2015).

Another crucial Argonaute interactor of dFmr1 is Piwi. A recent study from Jiang et al. in 2016 reported that dFmr1 and Piwi are present in the same complex in ovarian extracts and act together in the piRNA-mediated transcriptional silencing on the transposable elements in both somatic and germline tissues of the *Drosophila* ovary (Jiang et al., 2016). *dFmr1* mutations also influence the amount of a specific piRNA regulating the *roo* transposable elements. The N-terminal region of dFmr1, where the Tudor/Agenet domain is present (Ramos et al., 2006; Adams-Cioaba et al., 2010; Bozzetti et al., 2015; Iwasaki et al., 2015), is required for the interaction with Piwi.

Finally, no interaction has been reported between dFmr1 and mammalian FMRP with Ago3, another Argonaute protein that operates in the biogenesis of piRNAs in combination with Aubergine (Li et al., 2009).

### piRNA-Related dFmr1 Interactors Other Than Argonaute Proteins

The role of dFmr1 in the piRNA pathway is supported by its interaction with other components of the piRNA pathway, including Vasa, which is considered a molecular platform for the key components of the piRNA machinery, the so-called Amplifier complex (Xiol et al., 2014; Specchia et al., 2017). Figure 4 shows the colocalization of dFmr1 and Vasa at the nuage in testes, in particular at the piNG bodies. Emblematic examples have been described above where the direct interaction with four Argonaute proteins has been reported.

The zinc finger protein RP-8 (Zfrp8) also stands out as a very interesting interactor of dFmr1, even though its role in the piRNA pathway or in the human syndrome is still poorly understood.

Zfrp8 was initially identified for its fundamental role in the lymph glands, the site of larval hematopoiesis in *Drosophila* (Minakhina et al., 2007). In this tissue, Zfrp8 controls cell proliferation. Zfrp8 has also an essential role in follicle cells and in germline (Minakhina and Steward, 2010; Minakhina et al., 2014). This function is conserved during evolution, and the vertebrate Zfrp8 homolog, Pdcd2, is required for stem cell maintenance (Mu et al., 2010; Granier et al., 2014). Zfrp8 genetically interacts with several components of the piRNA pathway in the ovary including *vasa, ago3, spindle-E*, and *squash* (Stapleton et al., 2001; Pane et al., 2007; Li et al., 2009; Lasko, 2013; Tan et al., 2016). In addition, the distribution of Maelstrom, one of the known components of the piRNA pathway, is strongly affected in *Zfrp8* KD (Knock Down) ovaries and in germ stem cell (GSC) clones, in which the Zfrp8 protein had been silenced. The argument that strongly supports the role of Zfrp8 in the piRNA pathway is that its reduction affects the expression of the transposable elements in the ovaries (Minakhina et al., 2014), as also seen in animals, that are mutant for the member of the piRNA pathway. Notably, dFmr1 was found as a component of the Zfrp8 protein complex together with Nufip (nuclear FMRP interacting protein) and Trailer hitch (Tral) (Minakhina et al., 2014). Both these proteins were already identified as dFmr1 interactors: Nufip is one of the known interactors of FMRP in mammals (Bardoni et al., 2003), whereas Tral is a component of the RNP granules in *Drosophila* neurons (Barbee et al., 2006). Zfrp8 may have a role in the early assembly of ribosomes with translational repressors and, as a consequence, influences different processes during oogenesis, including transposons silencing (Tan et al., 2016). Very intriguingly, Hsp83, a known component of piRNA-mediated silencing pathway in the *Drosophila* gonads (Specchia et al., 2010; Gangaraju et al., 2011; Tan et al., 2016), was found in the Zfrp8 complex as well (Tan et al., 2016).

Finally, the TDP-43 protein involved in amyotrophic lateral sclerosis (ALS) also interacts with dFmr1. The physical association of these two proteins in ribonucleoproteic complexes was observed *in vivo*, in an ALS *Drosophila* model, and *in vitro*, in neuronal derived cells. FMRP deficit causes developmental defects and autistic behavior, whereas lack of TDP-43 leads to age-dependent neurodegeneration (Fallini et al., 2012; Yu et al., 2012; Coyne et al., 2015, 2017; Majumder et al., 2016). The unexpected link between TDP-43 and FMRP opens novel perspectives to understand the physiopathological mechanisms underlying these seemingly different pathologies.

### piRNA and TEs in the Nervous System

Although piRNAs were first identified in the gonads of mouse and *Drosophila* as regulators of transposable elements and repetitive sequences (Girard et al., 2006; Grivna et al., 2006; Vagin et al., 2006; Watanabe et al., 2006; Gunawardane et al., 2007; Nishida et al., 2007; Li et al., 2009; Malone et al., 2009), a specific set of piRNAs was found in the mouse hippocampus and in neuronal cultures (Lee et al., 2011). In addition, Ghildiyal et al. 2008) identified small RNA molecules in *Drosophila* heads displaying features resembling piRNAs (piRNA-like RNA molecules, pil-RNAs) (Ghildiyal et al., 2008). More recently, piRNAs with a role in the regulation of learning-related synaptic plasticity were also identified in the nervous system of *Aplysia* (Rajasethupathy et al., 2012). These discoveries represented the starting point for studies demonstrating the presence of piRNAs in somatic tissues and in particular in the brain of several organisms including *Drosophila* and humans (Baillie et al., 2011; Thomas et al., 2012; Perrat et al., 2013; Reilly et al., 2013; Ross et al., 2014; Weick and Miska, 2014). Furthermore, RNA-seq analyses revealed the presence of thousands of retrotransposon-derived piRNA-like molecules as well as the presence of factors, involved in the piRNA biogenesis, such as Mili and Maelstrom in hippocampal mammalian neurons. Mice lacking one or the other protein exhibit defects in locomotor activity and behavior (Matsumoto et al., 2015; Nandi et al., 2016). The presence of piRNAs in the nervous system suggests a role in the transposon silencing and hence in genome stability, which may impact on brain heterogeneity, aging, and also neurological diseases. Using different organisms, it was demonstrated that the deregulated expression of the transposable elements can induce their mobilization, which causes *de novo* insertions in the genome and hence triggers genomic variability in neuronal cells (Muotri et al., 2005; Coufal et al., 2009; Baillie et al., 2011; Evrony et al., 2012; Rajasethupathy et al., 2012; Perrat et al., 2013; Ross et al., 2014; Weick and Miska, 2014; Upton et al., 2015; Jachowicz et al., 2017).

Long-interspersed line-1 element (L1) is the only active element in the human genome (Beck et al., 2011) and can transpose in the neuronal precursor stem cells of the rat hippocampus. The new insertions were found in neuronal protein coding genes (Muotri et al., 2005). Engineered human L1 *in vitro* mobilization was also reported in neuronal precursor cells isolated from human fetal brains and embryonic stem cells. These discoveries strongly suggest that L1-mediated transposition has the potential to contribute to genotypic variation in neurons.

Whole genome sequencing and the analysis of the new insertions of a *gypsy*-construct support the idea that piRNA-mediated transposition also triggers cellular heterogeneity in the neurons of the *Drosophila* mushroom bodies, which are considered as the functional homolog of the mammalian hippocampus (Li et al., 2013; Perrat et al., 2013). The mobilization of the TEs occurs in a specific neuronal population, the αβ neurons, which contain a lower amount of Aub and Ago3 compared to the γδ neurons (Perrat et al., 2013), raising the concrete hypothesis that transposition may have a functional role in brain physiology. More recent data, however, do not seem to confirm the correlation between the increment in the expression of TEs and new integration sites in aging (Treiber and Waddell, 2017).

Clearly, the field is still very young and more studies will be required to firmly reach a consensus. However, even though the number of new genomic insertions does not exactly correlate with that expected from the remarkable increment of TE expression, a role of transposition in the nervous system must be considered, due to the growing amount of data on the topic.

Recent reports from many laboratories, conducted in *Drosophila*, in postmortem human tissues and in mammalian cells, support the relation between retrotransposition and neurological disorders (Muotri et al., 2010; Douville et al., 2011; Li et al., 2012; Tan et al., 2012; Rajan and Ramasamy, 2014; Krug et al., 2017; Morandi et al., 2017; Prudencio et al., 2017; Faulkner and Billon, 2018; Guo et al., 2018; Short et al., 2018). Significant examples are reported below. Parkinson’s disease (PD) is a neurodegenerative disorder that strongly affects movements. Aging represents a risk factor for the occurrence of sporadic PD (Martin, 2011). piRNAs and piRNA-like molecules are differentially expressed in “induced Pluripotent Stem Cells” (iPSCs) from patients during differentiation (Schulze et al., 2018).

Alzheimer disease (AD) is the neurodegenerative disorder that represents the most common cause of dementia. As a remarkable feature, the analysis of postmortem brains from Alzheimer patients reveals the presence of misfolded proteins, namely the β-amyloid peptide and the Tau protein. In addition, transposable elements are also deregulated in these tissues compared with normal brains and in adult brains of *Drosophila* expressing human Tau protein associated with AD (Qiu et al., 2017; Roy et al., 2017; Guo et al., 2018). Intriguingly, the Tau-induced neurological phenotypes can be partially rescued by manipulating DNA damage response key factors, providing a further link between transposition, genomic instability, and DNA (Guo et al., 2018).

Amyotrophic lateral sclerosis and frontotemporal dementia (FTD) are neurological disorders exhibiting a specific phenotypic spectrum causing dementia and cognitive impairment. They have been associated to a defect in TAR-DNA-binding protein 43 (TDP-43) (Douville et al., 2011; Li et al., 2015; Prudencio et al., 2017). Retrotransposition of one of the peculiar TEs with a functional similarity to viruses possessing also a “capsid,” whose name is *gypsy*, has been associated to ALS and FTD in a model expressing human TDP-43 (Krug et al., 2017). Even in the model of ALS, the modulation of DNA damage response (DDR) factors partially rescues the neurological phenotypes as occurs in Alzheimer’s disease model.

Finally, Fragile-X-associated tremor/ataxia syndrome (FXTAS) is a progressive neurological disorder associated to the premutation in the *Fmr1* gene reported before (expansion up to 90 RGG repeats in the regulatory region) (Amiri et al., 2008; Kronquist et al., 2008). Transgenic *Drosophila* lines that carry the FXTAS-associated expansion exhibit an increased expression of *gypsy*, hence providing the first link between the activation of transposons and neurodevelopmental disorders (Tan et al., 2012).

## DNA Damage Response and Fragile-X in *Drosophila* and Mammals

Damage to DNA can arise for different reasons and can generate multiple lesions including single- and double-strand breaks (SSBs and DSBs). These lesions set in motion the DNA repair machine that repairs the damage and prevents massive genome instability. This involves changes in the chromatin structure and cell cycle arrest.

Different factors are sequentially involved in the repairing process like the MRN complex, which is a eukaryotic protein complex consisting of Mre11, Rad 50 and Nbs1 proteins, followed by the ATM kinase, in turn phosphorylating several targets including p53 Chk2, BRCA1, and the key histone variant H2AX in mammals (Lou et al., 2006; Matsuoka et al., 2007; Lavin, 2008; Ciccia and Elledge, 2010). Proteins and processes participating in “DNA Damage Response” (DDR) cascade are conserved during evolution. In *Drosophila*, the majority of the information comes from studies on the meiotic checkpoint in ovaries, whose defects affect the fate of the embryonic dorsal cells (Ghabrial and Schupbach, 1999; Abdu et al., 2002; Staeva-Vieira et al., 2003; Cotta-Ramusino et al., 2011). Females displaying defects in this checkpoint process produce embryos with fused dorsal appendages and the mutations affect the so-called spindle class genes (Gonzalez-Reyes et al., 1997).

Interestingly, piRNA mutants also display defects in embryonic axis specification, which are thought to be a consequence of DNA damage mediated by the activation of transposable elements (Chen et al., 2007; Klattenhoff et al., 2007; Pane et al., 2007 Klattenhoff and Theurkauf, 2008). Mutations in *aub* and in other genes of the piRNA pathway such as *armitage* (Cook et al., 2004), *spindle-E* (Stapleton et al., 2001), *zucchini*, and *squash* (Pane et al., 2007), which belong to the spindle class genes, lead to the accumulation of the H2Av histone variant (Klattenhoff et al., 2007).

### DDR, Transposons, and Neurological Diseases

DNA lesions have been linked to neuronal decline in aging, oxidative stress conditions, and in neurological diseases (Ferrante et al., 1997; Adamec et al., 1999; Lu et al., 2004; Rass et al., 2007; Dobbin et al., 2013), even though the underlying molecular mechanisms remain poorly understood. Recently, the hyperactivation of the PARP-mediated DNA repair of single-strand breaks has been reported to be associated with neurodegeneration and ataxia in humans and mice (Nouspikel and Hanawalt, 2003; Katyal et al., 2014; Hoch et al., 2017).

As described above, transposable elements represent a considerable fraction of the eukaryotic genome and are regulated by the small RNA pathways, in particular the piRNA pathway. Defects in the small RNA-mediated regulation trigger their activation in the germline and in the somatic tissues of the *Drosophila* gonads, hence generating genome instability (Sarot et al., 2004; Kalmykova et al., 2005; Vagin et al., 2006; Chen et al., 2007; Pane et al., 2007; Specchia et al., 2010; Piacentini et al., 2014). A strong correlation between transposon mobilization and the DNA damage response also exists in human cells where the insertion of the Line-1 non-LTR retrotransposon depends on the DNA repair machine (Belgnaoui et al., 2006; Gasior et al., 2006). In addition, enhanced L1 mobilization has been reported in ataxia telangiectasia, a neurological disorder due to mutations in the ATM gene implicated in DNA repair (Coufal et al., 2011). These observations, linking the transposable elements and the DNA damage response, have led to the hypothesis that DNA breaks accumulate in piRNA mutants, where the transposons are massively activated (Klattenhoff and Theurkauf, 2008). This opens novel perspectives in understanding the causes of devastating neurological diseases, which, in the long term, will result in better therapeutical targets.

### DDR Has a Physiological Role in Neuronal Development

Emerging evidence support the hypothesis that activation of the DDR mediated by the double-strand breaks plays a physiological role in neuronal activity, by promoting the expression of the so-called early response genes in mice (Madabhushi et al., 2015). In neurons, the “early-response genes” code for transcription factors that are activated soon after the stimulation and regulate the cellular response by activating the expression of the “late response genes” (West and Greenberg, 2011). The “early” genes play a key role in synapse development and maturation and are hence required for learning and memory (Perez-Cadahia et al., 2011). Madabhushi et al. 2015) demonstrated that DSBs occur after neuronal activity at the transcriptional start sites of the early genes (and are related to the TopoII β activity). This facilitates the rapid response of these genes, whose promoters are bound to the “paused” RNA pol II in basal condition, that is, in the absence of stimuli (Kim et al., 2010). It is interesting to note that RNA pol II pausing is also observed at the promoters of genes that are expressed in response to environmental stimuli, and these genes are targeted by the *Drosophila* “HSP90 chaperone” (Sawarkar et al., 2010). This finding represents an intriguing link among “early” gene activation, HSP90, and DNA breaks.

The activation/movement of the transposable elements in the nervous system may induce genome instability, which in turn could connect DDR machinery and synaptic activity.

### dFmr1/FMRP Has a Role in the DNA Damage Response

FMRP may have a crucial role in this scenario because it has been related to the DNA damage response. Liu and collaborators demonstrated that *dfmr1* mutant flies display disproportioned cell death, related to DNA breaks and to marked genome instability, upon inducing DNA lesions (Liu et al., 2012). dFmr1 and FMRP had been previously shown to regulate cell cycle progression and differentiation in the germline as well as in the brain (Epstein et al., 2009; Yang et al., 2009; Callan et al., 2010; Papoulas et al., 2010), exerting their function in the early DDR through its Agenet and KH domains (Zhang et al., 2014). Soon after this observation, a result in mouse also supported a role of FMRP in the DNA damage response, regulating H2Ax phosphorylation, BRCA complex formation, and accumulation in embryonic fibroblasts and in mouse spermatocyte (Alpatov et al., 2014). This role is thought to be independent of the canonical function in the translational control of mRNAs involved in the synaptic plasticity (Brown et al., 2001; O’Donnell and Warren, 2002; Bassell and Warren, 2008) and requires FMRP N-terminal Tudor/Agenet domain for its binding to the H3 histone (Alpatov et al., 2014). All these discoveries assign a role to FMRP/dFmr1 in the DDR cascade, identifying this multifaceted protein as a hub for multiple cellular processes. Clearly, one of the most exciting and difficult features of FMRP is the presence of multiple domains involved in a variety of molecular processes, from the nuclear localization domain, the RNA-binding domains, and the Tudor/Agenet domain. This implies that a single protein has distinct roles depending on its localization in the different subcellular compartments. Future efforts will aim at disentangling the diverse functions of this molecular “Swiss knife” in development and physiology.

## Conclusions and Future Perspectives

**Figure 5:**
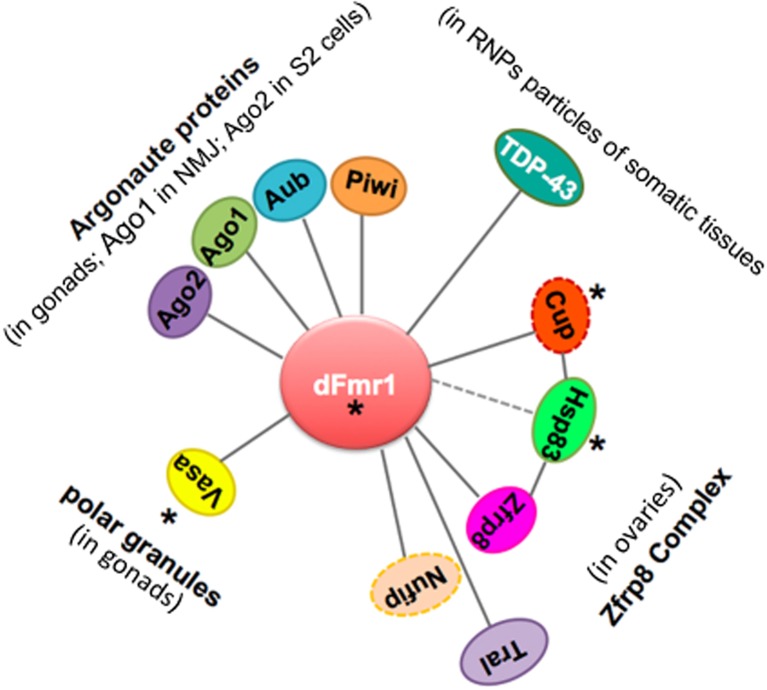
Scheme displaying the network of genetic and biochemical interactors of dFmr1 related to its role in genome stability. The tissues in which the genetic and/or biochemical interaction occurs are indicated (see text). Cup and Nufip are indicated by a dotted line, because they have not been yet tested for their role in the piRNA-mediated silencing of TEs. Hsp83 is connected to dFmr1 by a dotted line, because its interaction has not yet been demonstrated. Asterisks indicate the proteins that are part of the polar granules.

A growing number of studies report the identification of piRNAs, piRNA-related proteins, and piRNA-mediated transposition as key factors ensuring heterogeneity in mammalian neurons. Transposable elements are indeed emerging as novel players in neuronal development, and they may function through the DNA damage response pathway. In parallel, it has been shown that the *Drosophila* ortholog of the Fragile-X gene in humans, *dFmr1*, interacts with 4 of 5 Argonaute proteins in the gonads and in somatic tissues (Caudy et al., 2002; Ishizuka et al., 2002; Bozzetti et al., 2015; Jiang et al., 2016) and plays a role in the piRNA-mediated silencing of the repetitive sequences and transposon in the gonads (Bozzetti et al., 2015; Specchia et al., 2017). Figure 5 illustrates the potential role of dFmr1 in the protein network involved in genome stability. These discoveries open new perspectives for understanding the role and the mode of action of the dFmr1 protein in genome stability and pave the way to address its role in the piRNA pathway operating in the nervous system.

Key questions need now to be addressed: Does dFmr1 has a piRNA-mediated role in the brain and, if so, does its role in genome stability account for the multiple neurological phenotypes exhibited by *dFmr1* mutants and by the FraX patients? Typically, is the dFmr1 pathway linked to piRNAs involved in synaptic plasticity, learning and memory, and circadian behaviors? Should this role of dFmr1 be exerted in a specific temporal window during development as suggested by recent studies? (Weisz et al., 2015; Doll and Broadie, 2016; Doll et al., 2017).

*Drosophila* represents an attractive model for studying the Fragile-X syndrome and will help to address these questions because of the short generation time; the different types of genetic, cellular and molecular tools available; and the easy phenotype evaluation and rescue. *Drosophila melanogaster* offers a suitable *in vivo* model to prescreen numerous potential therapeutic molecules (McBride et al., 2005; Choi et al., 2010; Kanellopoulos et al., 2012; Hagerman et al., 2014), and clinical trials have been performed in human FraX patients, even though the results are not convincing. If the role of dFmr1 in the piRNA-mediated regulation of transposons is confirmed in the nervous system as well, new therapeutic possibility will open up. We are confident that dFmr1/FMRP will still surprise us and will help us in searching and finding potential therapeutical targets for the treatment of this devastating disease.

## Author Contributions

MB prepared the initial version of the manuscript. VS, SM, and AG significantly revised the manuscript. All authors provided intellectual contribution, edited, and approved the manuscript for publication in its complete version.

### Conflict of Interest Statement

The authors declare that the research was conducted in the absence of any commercial or financial relationships that could be construed as a potential conflict of interest.

## Footnotes

^1^http://www.hgmd.cf.ac.uk/ac/gene.php?gene=FMR1

## References

[ref1] Abdu U. Brodsky M. Schupbach T. (2002). Activation of a meiotic checkpoint during Drosophila oogenesis regulates the translation of Gurken through Chk2/Mnk. Curr. Biol. 12, 1645–1651. 10.1016/S0960-9822(02)01165-X, PMID: 12361566

[ref2] Adamec E. Vonsattel J. P. Nixon R. A. (1999). DNA strand breaks in Alzheimer’s disease. Brain Res. 849, 67–77. 10.1016/S0006-8993(99)02004-1, PMID: 10592288

[ref3] Adams-Cioaba M. A. Guo Y. Bian C. Amaya M. F. Lam R. Wasney G. A.. (2010). Structural studies of the tandem Tudor domains of fragile X mental retardation related proteins FXR1 and FXR2. PLoS One 5:e13559. 10.1371/journal.pone.0013559, PMID: 21072162 PMC2970552

[ref4] Aitken C. E. Lorsch J. R. (2012). A mechanistic overview of translation initiation in eukaryotes. Nat. Struct. Mol. Biol. 19, 568–576. 10.1038/nsmb.2303, PMID: 22664984

[ref5] Alpatov R. Lesch B. J. Nakamoto-Kinoshita M. Blanco A. Chen S. Stutzer A.. (2014). A chromatin-dependent role of the fragile X mental retardation protein FMRP in the DNA damage response. Cell 157, 869–881. 10.1016/j.cell.2014.03.040, PMID: 24813610 PMC4038154

[ref6] Amiri K. Hagerman R. J. Hagerman P. J. (2008). Fragile X-associated tremor/ataxia syndrome: an aging face of the fragile X gene. Arch. Neurol. 65, 19–25. 10.1001/archneurol.2007.30, PMID: 18195136

[ref7] Anand A. Kai T. (2012). The tudor domain protein kumo is required to assemble the nuage and to generate germline piRNAs in Drosophila. EMBO J. 31, 870–882. 10.1038/emboj.2011.449, PMID: 22157814 PMC3280549

[ref201] Aravin A. A. Hannon G. J. Brennecke J. (2007). The Piwi-piRNA pathway provides an adaptive defense in the transposon arms race. Science 318, 761–764. 10.1126/science.1146484, PMID: 17975059

[ref8] Aravin A. A. Lagos-Quintana M. Yalcin A. Zavolan M. Marks D. Snyder B.. (2003). The small RNA profile during Drosophila melanogaster development. Dev. Cell 5, 337–350. 10.1016/S1534-5807(03)00228-4, PMID: 12919683

[ref9] Aravin A. A. Naumova N. M. Tulin A. V. Vagin V. V. Rozovsky Y. M. Gvozdev V. A. (2001). Double-stranded RNA-mediated silencing of genomic tandem repeats and transposable elements in the *D. melanogaster* germline. Curr. Biol. 11, 1017–1027. 10.1016/S0960-9822(01)00299-8, PMID: 11470406

[ref10] Ascano M. Jr. Mukherjee N. Bandaru P. Miller J. B. Nusbaum J. D. Corcoran D. L.. (2012). FMRP targets distinct mRNA sequence elements to regulate protein expression. Nature 492, 382–386. 10.1038/nature11737, PMID: 23235829 PMC3528815

[ref11] Baillie J. K. Barnett M. W. Upton K. R. Gerhardt D. J. Richmond T. A. De Sapio F.. (2011). Somatic retrotransposition alters the genetic landscape of the human brain. Nature 479, 534–537. 10.1038/nature10531, PMID: 22037309 PMC3224101

[ref12] Barbee S. A. Estes P. S. Cziko A. M. Hillebrand J. Luedeman R. A. Coller J. M.. (2006). Staufen- and FMRP-containing neuronal RNPs are structurally and functionally related to somatic P bodies. Neuron 52, 997–1009. 10.1016/j.neuron.2006.10.028, PMID: 17178403 PMC1955741

[ref13] Bardoni B. Abekhoukh S. Zongaro S. Melko M. (2012). Intellectual disabilities, neuronal posttranscriptional RNA metabolism, and RNA-binding proteins: three actors for a complex scenario. Prog. Brain Res. 197, 29–51. 10.1016/B978-0-444-54299-1.00003-0 22541287

[ref14] Bardoni B. Willemsen R. Weiler I. J. Schenck A. Severijnen L. A. Hindelang C.. (2003). NUFIP1 (nuclear FMRP interacting protein 1) is a nucleocytoplasmic shuttling protein associated with active synaptoneurosomes. Exp. Cell Res. 289, 95–107. 10.1016/S0014-4827(03)00222-2, PMID: 12941608

[ref15] Bassell G. J. Warren S. T. (2008). Fragile X syndrome: loss of local mRNA regulation alters synaptic development and function. Neuron 60, 201–214. 10.1016/j.neuron.2008.10.004, PMID: 18957214 PMC3691995

[ref16] Bear M. F. Huber K. M. Warren S. T. (2004). The mGluR theory of fragile X mental retardation. Trends Neurosci. 27, 370–377. 10.1016/j.tins.2004.04.009, PMID: 15219735

[ref17] Beck C. R. Garcia-Perez J. L. Badge R. M. Moran J. V. (2011). LINE-1 elements in structural variation and disease. Annu. Rev. Genomics Hum. Genet. 12, 187–215. 10.1146/annurev-genom-082509-141802, PMID: 21801021 PMC4124830

[ref18] Belgnaoui S. M. Gosden R. G. Semmes O. J. Haoudi A. (2006). Human LINE-1 retrotransposon induces DNA damage and apoptosis in cancer cells. Cancer Cell Int. 6:13. 10.1186/1475-2867-6-13, PMID: 16670018 PMC1464142

[ref19] Belloni M. Tritto P. Bozzetti M. P. Palumbo G. Robbins L. G. (2002). Does stellate cause meiotic drive in Drosophila melanogaster? Genetics 161, 1551–1559. PMID: 12196400 10.1093/genetics/161.4.1551 PMC1462201

[ref20] Bolduc F. V. Bell K. Cox H. Broadie K. S. Tully T. (2008). Excess protein synthesis in Drosophila fragile X mutants impairs long-term memory. Nat. Neurosci. 11, 1143–1145. 10.1038/nn.2175, PMID: 18776892 PMC3038669

[ref21] Bolduc F. V. Bell K. Rosenfelt C. Cox H. Tully T. (2010). Fragile x mental retardation 1 and filamin a interact genetically in Drosophila long-term memory. Front. Neural Circuits 3:22. 10.3389/neuro.04.022.2009, PMID: 20190856 PMC2813723

[ref22] Bozzetti M. P. Fanti L. Di Tommaso S. Piacentini L. Berloco M. Tritto P.. (2012). The “Special” crystal-stellate system in Drosophila melanogaster reveals mechanisms underlying piRNA pathway-mediated canalization. Genet. Res. Int. 2012:324293. 10.1155/2012/324293, PMID: 22567384 PMC3335654

[ref23] Bozzetti M. P. Massari S. Finelli P. Meggio F. Pinna L. A. Boldyreff B.. (1995). The Ste locus, a component of the parasitic cry-Ste system of Drosophila melanogaster, encodes a protein that forms crystals in primary spermatocytes and mimics properties of the beta subunit of casein kinase 2. Proc. Natl. Acad. Sci. U. S. A. 92, 6067–6071. 10.1073/pnas.92.13.6067, PMID: 7597082 PMC41643

[ref24] Bozzetti M. P. Specchia V. Cattenoz P. B. Laneve P. Geusa A. Sahin H. B.. (2015). The Drosophila fragile X mental retardation protein participates in the piRNA pathway. J. Cell Sci. 128, 2070–2084. 10.1242/jcs.161810, PMID: 25908854

[ref25] Brennecke J. Aravin A. A. Stark A. Dus M. Kellis M. Sachidanandam R.. (2007). Discrete small RNA-generating loci as master regulators of transposon activity in Drosophila. Cell 128, 1089–1103. 10.1016/j.cell.2007.01.043, PMID: 17346786

[ref26] Brown V. Jin P. Ceman S. Darnell J. C. O’Donnell W. T. Tenenbaum S. A.. (2001). Microarray identification of FMRP-associated brain mRNAs and altered mRNA translational profiles in fragile X syndrome. Cell 107, 477–487. 10.1016/S0092-8674(01)00568-2, PMID: 11719188

[ref27] Callan M. A. Cabernard C. Heck J. Luois S. Doe C. Q. Zarnescu D. C. (2010). Fragile X protein controls neural stem cell proliferation in the Drosophila brain. Hum. Mol. Genet. 19, 3068–3079. 10.1093/hmg/ddq213, PMID: 20504994 PMC2901145

[ref29] Caudy A. A. Myers M. Hannon G. J. Hammond S. M. (2002). Fragile X-related protein and VIG associate with the RNA interference machinery. Genes Dev. 16, 2491–2496. 10.1101/gad.1025202, PMID: 12368260 PMC187452

[ref30] Chen Y. Pane A. Schupbach T. (2007). Cutoff and aubergine mutations result in retrotransposon upregulation and checkpoint activation in Drosophila. Curr. Biol. 17, 637–642. 10.1016/j.cub.2007.02.027, PMID: 17363252 PMC1905832

[ref31] Choi C. H. McBride S. M. Schoenfeld B. P. Liebelt D. A. Ferreiro D. Ferrick N. J.. (2010). Age-dependent cognitive impairment in a Drosophila fragile X model and its pharmacological rescue. Biogerontology 11, 347–362. 10.1007/s10522-009-9259-6, PMID: 20039205 PMC2866528

[ref32] Ciccia A. Elledge S. J. (2010). The DNA damage response: making it safe to play with knives. Mol. Cell 40, 179–204. 10.1016/j.molcel.2010.09.019, PMID: 20965415 PMC2988877

[ref33] Coffee R. L. Jr. Tessier C. R. Woodruff E. A. 3rd Broadie K. (2010). Fragile X mental retardation protein has a unique, evolutionarily conserved neuronal function not shared with FXR1P or FXR2P. Dis. Model. Mech. 3, 471–485. 10.1242/dmm.004598, PMID: 20442204 PMC2898537

[ref34] Cook H. A. Koppetsch B. S. Wu J. Theurkauf W. E. (2004). The Drosophila SDE3 homolog armitage is required for oskar mRNA silencing and embryonic axis specification. Cell 116, 817–829. 10.1016/S0092-8674(04)00250-8, PMID: 15035984

[ref35] Costa A. Wang Y. Dockendorff T. C. Erdjument-Bromage H. Tempst P. Schedl P.. (2005). The Drosophila fragile X protein functions as a negative regulator in the orb autoregulatory pathway. Dev. Cell 8, 331–342. 10.1016/j.devcel.2005.01.011, PMID: 15737929

[ref36] Cotta-Ramusino C. McDonald E. R. 3rd Hurov K. Sowa M. E. Harper J. W. Elledge S. J. (2011). A DNA damage response screen identifies RHINO, a 9-1-1 and TopBP1 interacting protein required for ATR signaling. Science 332, 1313–1317. 10.1126/science.1203430, PMID: 21659603 PMC4357496

[ref37] Coufal N. G. Garcia-Perez J. L. Peng G. E. Marchetto M. C. Muotri A. R. Mu Y.. (2011). Ataxia telangiectasia mutated (ATM) modulates long interspersed element-1 (L1) retrotransposition in human neural stem cells. Proc. Natl. Acad. Sci. U. S. A. 108, 20382–20387. 10.1073/pnas.1100273108, PMID: 22159035 PMC3251057

[ref38] Coufal N. G. Garcia-Perez J. L. Peng G. E. Yeo G. W. Mu Y. Lovci M. T.. (2009). L1 retrotransposition in human neural progenitor cells. Nature 460, 1127–1131. 10.1038/nature08248, PMID: 19657334 PMC2909034

[ref39] Coyne A. N. Lorenzini I. Chou C. C. Torvund M. Rogers R. S. Starr A.. (2017). Post-transcriptional inhibition of Hsc70-4/HSPA8 expression leads to synaptic vesicle cycling defects in multiple models of ALS. Cell Rep. 21, 110–125. 10.1016/j.celrep.2017.09.028, PMID: 28978466 PMC5679478

[ref40] Coyne A. N. Yamada S. B. Siddegowda B. B. Estes P. S. Zaepfel B. L. Johannesmeyer J. S.. (2015). Fragile X protein mitigates TDP-43 toxicity by remodeling RNA granules and restoring translation. Hum. Mol. Genet. 24, 6886–6898. 10.1093/hmg/ddv389, PMID: 26385636 PMC5007633

[ref41] Cziko A. M. McCann C. T. Howlett I. C. Barbee S. A. Duncan R. P. Luedemann R. (2009). Genetic modifiers of dFMR1 encode RNA granule components in Drosophila. Genetics 182, 1051–1060. 10.1534/genetics.109.103234, PMID: 19487564 PMC2728847

[ref42] Darnell J. C. Fraser C. E. Mostovetsky O. Stefani G. Jones T. A. Eddy S. R.. (2005). Kissing complex RNAs mediate interaction between the Fragile-X mental retardation protein KH2 domain and brain polyribosomes. Genes Dev. 19, 903–918. 10.1101/gad.1276805, PMID: 15805463 PMC1080130

[ref43] Darnell J. C. Van Driesche S. J. Zhang C. Hung K. Y. Mele A. Fraser C. E.. (2011). FMRP stalls ribosomal translocation on mRNAs linked to synaptic function and autism. Cell 146, 247–261. 10.1016/j.cell.2011.06.013, PMID: 21784246 PMC3232425

[ref44] Dobbin M. M. Madabhushi R. Pan L. Chen Y. Kim D. Gao J.. (2013). SIRT1 collaborates with ATM and HDAC1 to maintain genomic stability in neurons. Nat. Neurosci. 16, 1008–1015. 10.1038/nn.3460, PMID: 23852118 PMC4758134

[ref45] Dockendorff T. C. Labrador M. (2019). The Fragile X protein and genome function. Mol. Neurobiol. 56, 711–721. 10.1007/s12035-018-1122-9 29796988

[ref46] Dockendorff T. C. Su H. S. McBride S. M. Yang Z. Choi C. H. Siwicki K. K.. (2002). Drosophila lacking dfmr1 activity show defects in circadian output and fail to maintain courtship interest. Neuron 34, 973–984. 10.1016/S0896-6273(02)00724-9, PMID: 12086644

[ref47] Doll C. A. Broadie K. (2016). Neuron class-specific requirements for Fragile X mental retardation protein in critical period development of calcium signaling in learning and memory circuitry. Neurobiol. Dis. 89, 76–87. 10.1016/j.nbd.2016.02.006, PMID: 26851502 PMC4785039

[ref48] Doll C. A. Vita D. J. Broadie K. (2017). Fragile X mental retardation protein requirements in activity-dependent critical period neural circuit refinement. Curr. Biol. 27, 2318–2330 e3. 10.1016/j.cub.2017.06.046, PMID: 28756946 PMC5572839

[ref49] Douville R. Liu J. Rothstein J. Nath A. (2011). Identification of active loci of a human endogenous retrovirus in neurons of patients with amyotrophic lateral sclerosis. Ann. Neurol. 69, 141–151. 10.1002/ana.22149, PMID: 21280084 PMC3052883

[ref50] Drozd M. Bardoni B. Capovilla M. (2018). Modeling Fragile X syndrome in Drosophila. Front. Mol. Neurosci. 11:124. 10.3389/fnmol.2018.00124 29713264 PMC5911982

[ref51] Egorova K. S. Olenkina O. M. Kibanov M. V. Kalmykova A. I. Gvozdev V. A. Olenina L. V. (2009). Genetically derepressed nucleoplasmic stellate protein in spermatocytes of *D. melanogaster* interacts with the catalytic subunit of protein kinase 2 and carries histone-like lysine-methylated mark. J. Mol. Biol. 389, 895–906. 10.1016/j.jmb.2009.04.064, PMID: 19422836

[ref52] Epstein A. M. Bauer C. R. Ho A. Bosco G. Zarnescu D. C. (2009). Drosophila Fragile X protein controls cellular proliferation by regulating cbl levels in the ovary. Dev. Biol. 330, 83–92. 10.1016/j.ydbio.2009.03.011, PMID: 19306863

[ref53] Evrony G. D. Cai X. Lee E. Hills L. B. Elhosary P. C. Lehmann H. S.. (2012). Single-neuron sequencing analysis of L1 retrotransposition and somatic mutation in the human brain. Cell 151, 483–496. 10.1016/j.cell.2012.09.035, PMID: 23101622 PMC3567441

[ref54] Fallini C. Bassell G. J. Rossoll W. (2012). The ALS disease protein TDP-43 is actively transported in motor neuron axons and regulates axon outgrowth. Hum. Mol. Genet. 21, 3703–3718. 10.1093/hmg/dds205, PMID: 22641816 PMC3406762

[ref55] Faulkner G. J. Billon V. (2018). L1 retrotransposition in the soma: a field jumping ahead. Mob. DNA 9:22. 10.1186/s13100-018-0128-1 30002735 PMC6035798

[ref56] Feng Y. Absher D. Eberhart D. E. Brown V. Malter H. E. Warren S. T. (1997). FMRP associates with polyribosomes as an mRNP, and the I304N mutation of severe fragile X syndrome abolishes this association. Mol. Cell 1, 109–118. 10.1016/S1097-2765(00)80012-X, PMID: 9659908

[ref57] Ferrante R. J. Browne S. E. Shinobu L. A. Bowling A. C. Baik M. J. MacGarvey U.. (1997). Evidence of increased oxidative damage in both sporadic and familial amyotrophic lateral sclerosis. J. Neurochem. 69, 2064–2074. 10.1046/j.1471-4159.1997.69052064.x, PMID: 9349552

[ref58] Gangaraju V. K. Yin H. Weiner M. M. Wang J. Huang X. A. Lin H. (2011). Drosophila Piwi functions in Hsp90-mediated suppression of phenotypic variation. Nat. Genet. 43, 153–158. 10.1038/ng.743, PMID: 21186352 PMC3443399

[ref59] Gasior S. L. Wakeman T. P. Xu B. Deininger P. L. (2006). The human LINE-1 retrotransposon creates DNA double-strand breaks. J. Mol. Biol. 357, 1383–1393. 10.1016/j.jmb.2006.01.089, PMID: 16490214 PMC4136747

[ref60] Ghabrial A. Schupbach T. (1999). Activation of a meiotic checkpoint regulates translation of Gurken during Drosophila oogenesis. Nat. Cell Biol. 1, 354–357. 10.1038/14046, PMID: 10559962

[ref61] Ghildiyal M. Seitz H. Horwich M. D. Li C. Du T. Lee S.. (2008). Endogenous siRNAs derived from transposons and mRNAs in Drosophila somatic cells. Science 320, 1077–1081. 10.1126/science.1157396, PMID: 18403677 PMC2953241

[ref62] Girard A. Sachidanandam R. Hannon G. J. Carmell M. A. (2006). A germline-specific class of small RNAs binds mammalian Piwi proteins. Nature 442, 199–202. 10.1038/nature04917, PMID: 16751776

[ref63] Greenblatt E. J. Spradling A. C. (2018). Fragile X mental retardation 1 gene enhances the translation of large autism-related proteins. Science 361, 709–712. 10.1126/science.aas9963, PMID: 30115809 PMC6905618

[ref64] Godler D. E. Tassone F. Loesch D. Z. Taylor A. K. Gehling F. Hagerman R. J.. (2010). Methylation of novel markers of fragile X alleles is inversely correlated with FMRP expression and FMR1 activation ratio. Hum. Mol. Genet. 19, 1618–1632. 10.1093/hmg/ddq037, PMID: 20118148 PMC2846165

[ref65] Gonzalez-Reyes A. Elliott H. St Johnston D. (1997). Oocyte determination and the origin of polarity in Drosophila: the role of the spindle genes. Development 124, 4927–4937. 9362456 10.1242/dev.124.24.4927

[ref66] Granier C. J. Wang W. Tsang T. Steward R. Sabaawy H. E. Bhaumik M.. (2014). Conditional inactivation of PDCD2 induces p53 activation and cell cycle arrest. Biol. Open 3, 821–831. 10.1242/bio.20148326, PMID: 25150276 PMC4163659

[ref67] Grivna S. T. Beyret E. Wang Z. Lin H. (2006). A novel class of small RNAs in mouse spermatogenic cells. Genes Dev. 20, 1709–1714. 10.1101/gad.1434406, PMID: 16766680 PMC1522066

[ref68] Gunawardane L. S. Saito K. Nishida K. M. Miyoshi K. Kawamura Y. Nagami T.. (2007). A slicer-mediated mechanism for repeat-associated siRNA 5′end formation in Drosophila. Science 315, 1587–1590. 10.1126/science.1140494, PMID: 17322028

[ref69] Guo C. Jeong H. H. Hsieh Y. C. Klein H. U. Bennett D. A. De Jager P. L.. (2018). Tau activates transposable elements in Alzheimer’s disease. Cell Rep. 23, 2874–2880. 10.1016/j.celrep.2018.05.004, PMID: 29874575 PMC6181645

[ref70] Hagerman R. J. Des-Portes V. Gasparini F. Jacquemont S. Gomez-Mancilla B. (2014). Translating molecular advances in fragile X syndrome into therapy: a review. J. Clin. Psychiatry 75, e294–e307. 10.4088/JCP.13r08714, PMID: 24813413

[ref71] Hamilton S. M. Green J. R. Veeraragavan S. Yuva L. McCoy A. Wu Y.. (2014). Fmr1 and Nlgn3 knockout rats: novel tools for investigating autism spectrum disorders. Behav. Neurosci. 128, 103–109. 10.1037/a0035988, PMID: 24773431

[ref72] Hoch N. C. Hanzlikova H. Rulten S. L. Tetreault M. Komulainen E. Ju L.. (2017). XRCC1 mutation is associated with PARP1 hyperactivation and cerebellar ataxia. Nature 541, 87–91. 10.1038/nature20790, PMID: 28002403 PMC5218588

[ref73] Huber K. M. Gallagher S. M. Warren S. T. Bear M. F. (2002). Altered synaptic plasticity in a mouse model of fragile X mental retardation. Proc. Natl. Acad. Sci. U. S. A. 99, 7746–7750. 10.1073/pnas.122205699 12032354 PMC124340

[ref74] Hunter J. Rivero-Arias O. Angelov A. Kim E. Fotheringham I. Leal J. (2014). Epidemiology of fragile X syndrome: a systematic review and meta-analysis. Am. J. Med. Genet. A 164A, 1648–1658. 10.1002/ajmg.a.36511, PMID: 24700618

[ref75] Kanellopoulos A. K. Semelidou O. Kotini A. G. Anezaki M. Skoulakis E. M. (2012). Learning and memory deficits consequent to reduction of the fragile X mental retardation protein result from metabotropic glutamate receptor-mediated inhibition of cAMP signaling in Drosophila. J. Neurosci. 32, 13111–13124. 10.1523/JNEUROSCI.1347-12.2012, PMID: 22993428 PMC6621471

[ref76] Ishizuka A. Siomi M. C. Siomi H. (2002). A Drosophila fragile X protein interacts with components of RNAi and ribosomal proteins. Genes Dev. 16, 2497–2508. 10.1101/gad.1022002, PMID: 12368261 PMC187455

[ref77] Iwasaki Y. W. Siomi M. C. Siomi H. (2015). PIWI-Interacting RNA: its biogenesis and functions. Annu. Rev. Biochem. 84, 405–433. 10.1146/annurev-biochem-060614-034258, PMID: 25747396

[ref78] Jachowicz J. W. Bing X. Pontabry J. Boskovic A. Rando O. J. Torres-Padilla M. E. (2017). LINE-1 activation after fertilization regulates global chromatin accessibility in the early mouse embryo. Nat. Genet. 49, 1502–1510. 10.1038/ng.3945, PMID: 28846101

[ref79] Jiang F. Lu F. Li P. Liu W. Zhao L. Wang Q. (2016). Drosophila homolog of FMRP maintains genome integrity by interacting with Piwi. Int. J. Genet. Genomics 43, 11–24. 10.1016/j.jgg.2015.11.001 26842990

[ref80] Jin P. Zarnescu D. C. Ceman S. Nakamoto M. Mowrey J. Jongens T. A.. (2004). Biochemical and genetic interaction between the fragile X mental retardation protein and the microRNA pathway. Nat. Neurosci. 7, 113–117. 10.1038/nn1174, PMID: 14703574

[ref81] Johannisson R. Rehder H. Wendt V. Schwinger E. (1987). Spermatogenesis in two patients with the fragile X syndrome. I. Histology: light and electron microscopy. Hum. Genet. 76, 141–147. 10.1007/BF00284911, PMID: 3610145

[ref82] Kalmykova A. I. Klenov M. S. Gvozdev V. A. (2005). Argonaute protein PIWI controls mobilization of retrotransposons in the Drosophila male germline. Nucleic Acids Res. 33, 2052–2059. 10.1093/nar/gki323, PMID: 15817569 PMC1074743

[ref83] Katyal S. Lee Y. Nitiss K. C. Downing S. M. Li Y. Shimada M.. (2014). Aberrant topoisomerase-1 DNA lesions are pathogenic in neurodegenerative genome instability syndromes. Nat. Neurosci. 17, 813–821. 10.1038/nn.3715, PMID: 24793032 PMC4074009

[ref84] Kibanov M. V. Egorova K. S. Ryazansky S. S. Sokolova O. A. Kotov A. A. Olenkina O. M. (2011). A novel organelle, the piNG-body, in the nuage of Drosophila male germ cells is associated with piRNA-mediated gene silencing. Mol. Biol. Cell 22, 3410–3419. 10.1091/mbc.E11-02-0168 21775629 PMC3172265

[ref85] Kim H. Erickson B. Luo W. Seward D. Graber J. H. Pollock D. D.. (2010). Gene-specific RNA polymerase II phosphorylation and the CTD code. Nat. Struct. Mol. Biol. 17, 1279–1286. 10.1038/nsmb.1913, PMID: 20835241 PMC3048030

[ref86] Klattenhoff C. Bratu D. P. McGinnis-Schultz N. Koppetsch B. S. Cook H. A. Theurkauf W. E. (2007). Drosophila rasiRNA pathway mutations disrupt embryonic axis specification through activation of an ATR/Chk2 DNA damage response. Dev. Cell 12, 45–55. 10.1016/j.devcel.2006.12.001, PMID: 17199040

[ref87] Klattenhoff C. Theurkauf W. (2008). Biogenesis and germline functions of piRNAs. Development 135, 3–9. 10.1242/dev.006486, PMID: 18032451

[ref88] Kronquist K. E. Sherman S. L. Spector E. B. (2008). Clinical significance of tri-nucleotide repeats in Fragile X testing: a clarification of American College of Medical Genetics guidelines. Genet. Med. Off. J. Am. Coll. Med. Genet. 10, 845–847. 10.1097/GIM.0b013e31818b0c8a, PMID: 18941415 PMC3111547

[ref89] Krug L. Chatterjee N. Borges-Monroy R. Hearn S. Liao W. W. Morrill K.. (2017). Retrotransposon activation contributes to neurodegeneration in a Drosophila TDP-43 model of ALS. PLoS Genet. 13:e1006635. 10.1371/journal.pgen.1006635, PMID: 28301478 PMC5354250

[ref90] Lasko P. (2013). The DEAD-box helicase Vasa: evidence for a multiplicity of functions in RNA processes and developmental biology. Biochim. Biophys. Acta 1829, 810–816. 10.1016/j.bbagrm.2013.04.005 23587717

[ref91] Lavin M. F. (2008). Ataxia-telangiectasia: from a rare disorder to a paradigm for cell signalling and cancer. Nat. Rev. Mol. Cell Biol. 9, 759–769. 10.1038/nrm2514, PMID: 18813293

[ref92] Lee E. J. Banerjee S. Zhou H. Jammalamadaka A. Arcila M. Manjunath B. S.. (2011). Identification of piRNAs in the central nervous system. RNA 17, 1090–1099. 10.1261/rna.2565011, PMID: 21515829 PMC3096041

[ref93] Lee J. Peng Y. Lin W. Y. Parrish J. Z. (2015). Coordinate control of terminal dendrite patterning and dynamics by the membrane protein Raw. Development 142, 162–173. 10.1242/dev.113423 25480915 PMC4299136

[ref202] Li W. Prazak L. Chatterjee N. Gruninger S. Krug L. Theodorou D. (2013). Activation of transposable elements during aging and neuronal decline in Drosophila. Nature Neuroscience 16, 529–531. 10.1038/nn.3368, PMID: 23563579 PMC3821974

[ref94] Li C. Vagin V. V. Lee S. Xu J. Ma S. Xi H.. (2009). Collapse of germline piRNAs in the absence of Argonaute3 reveals somatic piRNAs in flies. Cell 137, 509–521. 10.1016/j.cell.2009.04.027, PMID: 19395009 PMC2768572

[ref95] Li W. Jin Y. Prazak L. Hammell M. Dubnau J. (2012). Transposable elements in TDP-43-mediated neurodegenerative disorders. PLoS One 7:e44099. 10.1371/journal.pone.0044099, PMID: 22957047 PMC3434193

[ref96] Li W. Lee M. H. Henderson L. Tyagi R. Bachani M. Steiner J.. (2015). Human endogenous retrovirus-K contributes to motor neuron disease. Sci. Transl. Med. 7:307ra153. 10.1126/scitranslmed.aac8201, PMID: 26424568 PMC6344353

[ref97] Liu W. Jiang F. Bi X. Zhang Y. Q. (2012). Drosophila FMRP participates in the DNA damage response by regulating G2/M cell cycle checkpoint and apoptosis. Hum. Mol. Genet. 21, 4655–4568. 10.1093/hmg/dds307, PMID: 22843500

[ref98] Livak K. J. (1984). Organization and mapping of a sequence on the Drosophila melanogaster X and Y chromosomes that is transcribed during spermatogenesis. Genetics 107, 611–634. PMID: 6430749 10.1093/genetics/107.4.611 PMC1202380

[ref99] Lou Z. Minter-Dykhouse K. Franco S. Gostissa M. Rivera M. A. Celeste A.. (2006). MDC1 maintains genomic stability by participating in the amplification of ATM-dependent DNA damage signals. Mol. Cell 21, 187–200. 10.1016/j.molcel.2005.11.025, PMID: 16427009

[ref100] Lu T. Pan Y. Kao S. Y. Li C. Kohane I. Chan J.. (2004). Gene regulation and DNA damage in the ageing human brain. Nature 429, 883–891. 10.1038/nature02661, PMID: 15190254

[ref101] Madabhushi R. Gao F. Pfenning A. R. Pan L. Yamakawa S. Seo J.. (2015). Activity-induced DNA breaks govern the expression of neuronal early-response genes. Cell 161, 1592–1605. 10.1016/j.cell.2015.05.032, PMID: 26052046 PMC4886855

[ref102] Majumder P. Chu J. F. Chatterjee B. Swamy K. B. Shen C. J. (2016). Co-regulation of mRNA translation by TDP-43 and Fragile X syndrome protein FMRP. Acta Neuropathol. 132, 721–738. 10.1007/s00401-016-1603-8, PMID: 27518042 PMC5073124

[ref103] Malone C. D. Brennecke J. Dus M. Stark A. McCombie W. R. Sachidanandam R.. (2009). Specialized piRNA pathways act in germline and somatic tissues of the Drosophila ovary. Cell 137, 522–535. 10.1016/j.cell.2009.03.040, PMID: 19395010 PMC2882632

[ref104] Martin F. C. (2011). Falls risk factors: assessment and management to prevent falls and fractures. Can. J. Aging 30, 33–44. 10.1017/S0714980810000747, PMID: 24650637

[ref105] Matsumoto N. Sato K. Nishimasu H. Namba Y. Miyakubi K. Dohmae N.. (2015). Crystal structure and activity of the endoribonuclease domain of the piRNA Pathway Factor Maelstrom. Cell Rep. 11, 366–375. 10.1016/j.celrep.2015.03.030, PMID: 25865890

[ref106] Matsuoka S. Ballif B. A. Smogorzewska A. McDonald E. R. 3rd Hurov K. E. Luo J.. (2007). ATM and ATR substrate analysis reveals extensive protein networks responsive to DNA damage. Science 316, 1160–1166. 10.1126/science.1140321, PMID: 17525332

[ref107] Maurin H. Chong S. A. Kraev I. Davies H. Kremer A. Seymour C. M.. (2014). Early structural and functional defects in synapses and myelinated axons in stratum lacunosum moleculare in two preclinical models for tauopathy. PLoS One 9:e87605. 10.1371/journal.pone.0087605, PMID: 24498342 PMC3912020

[ref108] McBride S. M. Bell A. J. Jongens T. A. (2012). Behavior in a Drosophila model of fragile X. Results Probl. Cell Differ. 54, 83–117. 22009349 10.1007/978-3-642-21649-7_6

[ref109] McBride S. M. Choi C. H. Wang Y. Liebelt D. Braunstein E. Ferreiro D.. (2005). Pharmacological rescue of synaptic plasticity, courtship behavior, and mushroom body defects in a Drosophila model of fragile X syndrome. Neuron 45, 753–764. 10.1016/j.neuron.2005.01.038, PMID: 15748850

[ref110] Minakhina S. Changela N. Steward R. (2014). Zfrp8/PDCD2 is required in ovarian stem cells and interacts with the piRNA pathway machinery. Development 141, 259–268. 10.1242/dev.101410 24381196 PMC3879809

[ref111] Minakhina S. Druzhinina M. Steward R. (2007). Zfrp8, the Drosophila ortholog of PDCD2, functions in lymph gland development and controls cell proliferation. Development 134, 2387–2396. 10.1242/dev.003616, PMID: 17522156

[ref112] Minakhina S. Steward R. (2010). Hematopoietic stem cells in Drosophila. Development 137, 27–31. 10.1242/dev.043943 20023157 PMC2796932

[ref113] Morales J. Hiesinger P. R. Schroeder A. J. Kume K. Verstreken P. Jackson F. R.. (2002). Drosophila fragile X protein, DFXR, regulates neuronal morphology and function in the brain. Neuron 34, 961–972. 10.1016/S0896-6273(02)00731-6, PMID: 12086643

[ref114] Morandi E. Tanasescu R. Tarlinton R. E. Constantinescu C. S. Zhang W. Tench C.. (2017). The association between human endogenous retroviruses and multiple sclerosis: a systematic review and meta-analysis. PLoS One 12:e0172415. 10.1371/journal.pone.0172415, PMID: 28207850 PMC5313176

[ref115] Mu W. Munroe R. J. Barker A. K. Schimenti J. C. (2010). PDCD2 is essential for inner cell mass development and embryonic stem cell maintenance. Dev. Biol. 347, 279–288. 10.1016/j.ydbio.2010.08.026, PMID: 20813103 PMC2957520

[ref116] Mugat B. Akkouche A. Serrano V. Armenise C. Li B. Brun C.. (2015). MicroRNA-dependent transcriptional silencing of transposable elements in Drosophila follicle cells. PLoS Genet. 11:e1005194. 10.1371/journal.pgen.1005194, PMID: 25993106 PMC4451950

[ref117] Muotri A. R. Chu V. T. Marchetto M. C. Deng W. Moran J. V. Gage F. H. (2005). Somatic mosaicism in neuronal precursor cells mediated by L1 retrotransposition. Nature 435, 903–910. 10.1038/nature03663, PMID: 15959507

[ref118] Muotri A. R. Marchetto M. C. Coufal N. G. Oefner R. Yeo G. Nakashima K.. (2010). L1 retrotransposition in neurons is modulated by MeCP2. Nature 468, 443–446. 10.1038/nature09544, PMID: 21085180 PMC3059197

[ref119] Nagao A. Sato K. Nishida K. M. Siomi H. Siomi M. C. (2011). Gender-specific hierarchy in nuage localization of PIWI-interacting RNA factors in Drosophila. Front. Genet. 2:55. 10.3389/fgene.2011.00055, PMID: 22303351 PMC3268608

[ref120] Nandi S. Chandramohan D. Fioriti L. Melnick A. M. Hebert J. M. Mason C. E.. (2016). Roles for small noncoding RNAs in silencing of retrotransposons in the mammalian brain. Proc. Natl. Acad. Sci. U. S. A. 113, 12697–12702. 10.1073/pnas.1609287113, PMID: 27791114 PMC5111663

[ref121] Napoli I. Mercaldo V. Boyl P. P. Eleuteri B. Zalfa F. De Rubeis S.. (2008). The fragile X syndrome protein represses activity-dependent translation through CYFIP1, a new 4E-BP. Cell 134, 1042–1054. 10.1016/j.cell.2008.07.031, PMID: 18805096

[ref122] Nishida K. M. Saito K. Mori T. Kawamura Y. Nagami-Okada T. Inagaki S.. (2007). Gene silencing mechanisms mediated by Aubergine piRNA complexes in Drosophila male gonad. RNA 13, 1911–1922. 10.1261/rna.744307, PMID: 17872506 PMC2040086

[ref123] Nouspikel T. Hanawalt P. C. (2003). When parsimony backfires: neglecting DNA repair may doom neurons in Alzheimer’s disease. BioEssays: News Rev. Mol. Cell. Dev. Biol. 25, 168–173. 10.1002/bies.10227, PMID: 12539243

[ref124] O’Donnell W. T. Warren S. T. (2002). A decade of molecular studies of fragile X syndrome. Annu. Rev. Neurosci. 25, 315–338. 10.1146/annurev.neuro.25.112701.142909, PMID: 12052912

[ref125] Okray Z. de Esch C. E. Van Esch H. Devriendt K. Claeys A. Yan J.. (2015). A novel fragile X syndrome mutation reveals a conserved role for the carboxy-terminus in FMRP localization and function. EMBO Mol. Med. 7, 423–437. 10.15252/emmm.201404576, PMID: 25693964 PMC4403044

[ref126] Palumbo G. Bonaccorsi S. Robbins L. G. Pimpinelli S. (1994). Genetic analysis of Stellate elements of Drosophila melanogaster. Genetics 138, 1181–1197. 7896100 10.1093/genetics/138.4.1181 PMC1206257

[ref127] Pan L. Zhang Y. Q. Woodruff E. Broadie K. (2004). The Drosophila fragile X gene negatively regulates neuronal elaboration and synaptic differentiation. Curr. Biol. 14, 1863–1870. 10.1016/j.cub.2004.09.085, PMID: 15498496

[ref128] Pane A. Wehr K. Schupbach T. (2007). Zucchini and squash encode two putative nucleases required for rasiRNA production in the Drosophila germline. Dev. Cell 12, 851–862. 10.1016/j.devcel.2007.03.022, PMID: 17543859 PMC1945814

[ref129] Papoulas O. Monzo K. F. Cantin G. T. Ruse C. Yates J. R. 3rd Ryu Y. H.. (2010). dFMRP and Caprin, translational regulators of synaptic plasticity, control the cell cycle at the Drosophila mid-blastula transition. Development 137, 4201–4209. 10.1242/dev.055046, PMID: 21068064 PMC2990211

[ref130] Patil V. S. Kai T. (2010). Repression of retroelements in Drosophila germline via piRNA pathway by the Tudor domain protein Tejas. Curr. Biol. 20, 724–730. 10.1016/j.cub.2010.02.046, PMID: 20362446

[ref131] Penagarikano O. Mulle J. G. Warren S. T. (2007). The pathophysiology of fragile x syndrome. Annu. Rev. Genomics Hum. Genet. 8, 109–129. 10.1146/annurev.genom.8.080706.092249, PMID: 17477822

[ref132] Perez-Cadahia B. Drobic B. Davie J. R. (2011). Activation and function of immediate-early genes in the nervous system. Biochem. Cell Biol. 89, 61–73. 10.1139/O10-138, PMID: 21326363

[ref133] Perrat P. N. DasGupta S. Wang J. Theurkauf W. Weng Z. Rosbash M.. (2013). Transposition-driven genomic heterogeneity in the Drosophila brain. Science 340, 91–95. 10.1126/science.1231965, PMID: 23559253 PMC3887341

[ref134] Piacentini L. Fanti L. Specchia V. Bozzetti M. P. Berloco M. Palumbo G.. (2014). Transposons, environmental changes, and heritable induced phenotypic variability. Chromosoma 123, 345–354. 10.1007/s00412-014-0464-y, PMID: 24752783 PMC4107273

[ref135] Pisa V. Cozzolino M. Gargiulo S. Ottone C. Piccioni F. Monti M.. (2009). The molecular chaperone Hsp90 is a component of the cap-binding complex and interacts with the translational repressor Cup during Drosophila oogenesis. Gene 432, 67–74. 10.1016/j.gene.2008.11.025, PMID: 19101615

[ref136] Prudencio M. Gonzales P. K. Cook C. N. Gendron T. F. Daughrity L. M. Song Y.. (2017). Repetitive element transcripts are elevated in the brain of C9orf72 ALS/FTLD patients. Hum. Mol. Genet. 26, 3421–3431. 10.1093/hmg/ddx233, PMID: 28637276 PMC5886204

[ref137] Qiu W. Guo X. Lin X. Yang Q. Zhang W. Zhang Y.. (2017). Transcriptome-wide piRNA profiling in human brains of Alzheimer’s disease. Neurobiol. Aging 57, 170–177. 10.1016/j.neurobiolaging.2017.05.020, PMID: 28654860 PMC5542056

[ref138] Quartier A. Poquet H. Gilbert-Dussardier B. Rossi M. Casteleyn A. S. Portes V. D.. (2017). Intragenic FMR1 disease-causing variants: a significant mutational mechanism leading to Fragile-X syndrome. Eur. J. Hum. Genet. 25, 423–431. 10.1038/ejhg.2016.204, PMID: 28176767 PMC5386424

[ref139] Rajan K. S. Ramasamy S. (2014). Retrotransposons and piRNA: the missing link in central nervous system. Neurochem. Int. 77, 94–102. 10.1016/j.neuint.2014.05.017, PMID: 24925769

[ref140] Rajasethupathy P. Antonov I. Sheridan R. Frey S. Sander C. Tuschl T.. (2012). A role for neuronal piRNAs in the epigenetic control of memory-related synaptic plasticity. Cell 149, 693–707. 10.1016/j.cell.2012.02.057, PMID: 22541438 PMC3442366

[ref141] Ramos A. Hollingworth D. Adinolfi S. Castets M. Kelly G. Frenkiel T. A.. (2006). The structure of the N-terminal domain of the fragile X mental retardation protein: a platform for protein-protein interaction. Structure 14, 21–31. 10.1016/j.str.2005.09.018, PMID: 16407062

[ref142] Rass U. Ahel I. West S. C. (2007). Defective DNA repair and neurodegenerative disease. Cell 130, 991–1004. 10.1016/j.cell.2007.08.043, PMID: 17889645

[ref143] Reeve S. P. Lin X. Sahin B. H. Jiang F. Yao A. Liu Z.. (2008). Mutational analysis establishes a critical role for the N terminus of fragile X mental retardation protein FMRP. J. Neurosci. 28, 3221–3226. 10.1523/JNEUROSCI.5528-07.2008, PMID: 18354025 PMC6670702

[ref144] Reilly M. T. Faulkner G. J. Dubnau J. Ponomarev I. Gage F. H. (2013). The role of transposable elements in health and diseases of the central nervous system. J. Neurosci. 33, 17577–17586. 10.1523/JNEUROSCI.3369-13.2013, PMID: 24198348 PMC3818539

[ref145] Ross R. J. Weiner M. M. Lin H. (2014). PIWI proteins and PIWI-interacting RNAs in the soma. Nature 505, 353–359. 10.1038/nature12987, PMID: 24429634 PMC4265809

[ref146] Rossetti R. Ferrari I. Bonomi M. Persani L. (2017). Genetics of primary ovarian insufficiency. Clin. Genet. 91, 183–198. 10.1007/s10815-014-0342-9, PMID: 27861765

[ref147] Roy J. Sarkar A. Parida S. Ghosh Z. Mallick B. (2017). Small RNA sequencing revealed dysregulated piRNAs in Alzheimer’s disease and their probable role in pathogenesis. Mol. BioSyst. 13, 565–576. 10.1039/c6mb00699j, PMID: 28127595

[ref148] Sahin H. B. Karatas O. F. Specchia V. Tommaso S. D. Diebold C. Bozzetti M. P.. (2016). Novel mutants of the aubergine gene. Fly 10, 81–90. 10.1080/19336934.2016.1174355, PMID: 27064345 PMC4934710

[ref149] Saito K. Ishizu H. Komai M. Kotani H. Kawamura Y. Nishida K. M.. (2010). Roles for the Yb body components Armitage and Yb in primary piRNA biogenesis in Drosophila. Genes Dev. 24, 2493–2498. 10.1101/gad.1989510, PMID: 20966047 PMC2975925

[ref150] Santoro M. R. Bray S. M. Warren S. T. (2012). Molecular mechanisms of fragile X syndrome: a twenty-year perspective. Annu. Rev. Pathol. 7, 219–245. 10.1146/annurev-pathol-011811-132457, PMID: 22017584

[ref151] Sarot E. Payen-Groschene G. Bucheton A. Pelisson A. (2004). Evidence for a piwi-dependent RNA silencing of the gypsy endogenous retrovirus by the Drosophila melanogaster flamenco gene. Genetics 166, 1313–1321. 10.1534/genetics.166.3.1313, PMID: 15082550 PMC1470774

[ref152] Sawarkar R. Sievers C. Paro R. (2010). HSP90 globally targets paused RNA Polymerase to regulate gene expression in response to environmental stimuli. Cell 149, 807–818. 10.1016/j.cell.2012.02.061 22579285

[ref153] Schenck A. Bardoni B. Langmann C. Harden N. Mandel J. L. Giangrande A. (2003). CYFIP/Sra-1 controls neuronal connectivity in Drosophila and links the Rac1 GTPase pathway to the fragile X protein. Neuron 38, 887–898. 10.1016/S0896-6273(03)00354-4, PMID: 12818175

[ref154] Schulze M. Sommer A. Plotz S. Farrell M. Winner B. Grosch J. (2018). Sporadic Parkinson’s disease derived neuronal cells show disease-specific mRNA and small RNA signatures with abundant deregulation of piRNAs. Acta Neuropathol. Commun. 6:58. 10.1186/s40478-018-0561-x 29986767 PMC6038190

[ref155] Sears J. C. Broadie K. (2017). Fragile X mental retardation protein regulates activity-dependent membrane trafficking and trans-synaptic signaling mediating synaptic remodeling. Front. Mol. Neurosci. 10:440. 10.3389/fnmol.2017.00440 29375303 PMC5770364

[ref156] Sehgal A. Price J. L. Man B. Young M. W. (1994). Loss of circadian behavioral rhythms and per RNA oscillations in the Drosophila mutant timeless. Science 263, 1603–1606. 10.1126/science.8128246, PMID: 8128246

[ref157] Short P. J. McRae J. F. Gallone G. Sifrim A. Won H. Geschwind D. H.. (2018). De novo mutations in regulatory elements in neurodevelopmental disorders. Nature 555, 611–616. 10.1038/nature25983, PMID: 29562236 PMC5912909

[ref158] Siomi M. C. Siomi H. Sauer W. H. Srinivasan S. Nussbaum R. L. Dreyfuss G. (1995). FXR1, an autosomal homolog of the fragile X mental retardation gene. EMBO J. 14, 2401–2408. 10.1002/j.1460-2075.1995.tb07237.x, PMID: 7781595 PMC398353

[ref159] Siomi M. C. Zhang Y. Siomi H. Dreyfuss G. (1996). Specific sequences in the fragile X syndrome protein FMR1 and the FXR proteins mediate their binding to 60S ribosomal subunits and the interactions among them. Mol. Cell. Biol. 16, 3825–3832. 10.1128/MCB.16.7.3825, PMID: 8668200 PMC231379

[ref160] Slegtenhorst-Eegdeman K. E. de Rooij D. G. Verhoef-Post M. van de Kant H. J. Bakker C. E. Oostra B. A.. (1998). Macroorchidism in FMR1 knockout mice is caused by increased Sertoli cell proliferation during testicular development. Endocrinology 139, 156–162. 10.1210/endo.139.1.5706, PMID: 9421410

[ref161] Specchia V. Benna C. Mazzotta G. M. Piccin A. Zordan M. A. Costa R.. (2008). Aubergine gene overexpression in somatic tissues of aubergine(sting) mutants interferes with the RNAi pathway of a yellow hairpin dsRNA in Drosophila melanogaster. Genetics 178, 1271–1282. 10.1534/genetics.107.078626, PMID: 18385112 PMC2278088

[ref162] Specchia V. Bozzetti M. P. (2009). Different aubergine alleles confirm the specificity of different RNAi pathways in Drosophila melanogaster. Fly 3, 170–172. PMID: 19242123 10.4161/fly.8054

[ref163] Specchia V. D’Attis S. Puricella A. Bozzetti M. P. (2017). dFmr1 plays roles in small RNA pathways of Drosophila melanogaster. Int. J. Mol. Sci. 18:E1066. 10.3390/ijms18051066, PMID: 28509881 PMC5454977

[ref164] Specchia V. Piacentini L. Tritto P. Fanti L. D’Alessandro R. Palumbo G.. (2010). Hsp90 prevents phenotypic variation by suppressing the mutagenic activity of transposons. Nature 463, 662–665. 10.1038/nature08739, PMID: 20062045

[ref165] Staeva-Vieira E. Yoo S. Lehmann R. (2003). An essential role of DmRad51/SpnA in DNA repair and meiotic checkpoint control. EMBO J. 22, 5863–5874. 10.1093/emboj/cdg564, PMID: 14592983 PMC275421

[ref166] Stapleton W. Das S. McKee B. D. (2001). A role of the Drosophila homeless gene in repression of Stellate in male meiosis. Chromosoma 110, 228–240. 10.1007/s004120100136, PMID: 11513298

[ref167] Suhl J. A. Warren S. T. (2015). Single-nucleotide mutations in FMR1 reveal novel functions and regulatory mechanisms of the Fragile X syndrome protein FMRP. J. Exp. Neurosci. 9, 35–41. 10.4137/JEN.S25524, PMID: 26819560 PMC4720182

[ref168] Szakmary A. Reedy M. Qi H. Lin H. (2009). The Yb protein defines a novel organelle and regulates male germline stem cell self-renewal in Drosophila melanogaster. J. Cell Biol. 185, 613–627. 10.1083/jcb.200903034, PMID: 19433453 PMC2711570

[ref169] Swanger S. A. Bassell G. J. (2011). Making and breaking synapses through local mRNA regulation. Curr. Opin. Genet. Dev. 21, 414–421. 10.1016/j.gde.2011.04.002, PMID: 21530231 PMC3149745

[ref170] Tamanini F. Meijer N. Verheij C. Willems P. J. Galjaard H. Oostra B. A.. (1996). FMRP is associated to the ribosomes via RNA. Hum. Mol. Genet. 5, 809–813. 10.1093/hmg/5.6.809, PMID: 8776596

[ref171] Tan H. Qurashi A. Poidevin M. Nelson D. L. Li H. Jin P. (2012). Retrotransposon activation contributes to fragile X premutation rCGG-mediated neurodegeneration. Hum. Mol. Genet. 21, 57–65. 10.1093/hmg/ddr437 21940752 PMC3235010

[ref172] Tan W. Schauder C. Naryshkina T. Minakhina S. Steward R. (2016). Zfrp8 forms a complex with fragile-X mental retardation protein and regulates its localization and function. Dev. Biol. 410, 202–212. 10.1016/j.ydbio.2015.12.008, PMID: 26772998 PMC4768487

[ref173] Tessier C. R. Broadie K. (2012). Molecular and genetic analysis of the Drosophila model of fragile X syndrome. Results Probl. Cell Differ. 54, 119–156. 22009350 10.1007/978-3-642-21649-7_7 PMC4936787

[ref174] Thomas C. A. Paquola A. C. Muotri A. R. (2012). LINE-1 retrotransposition in the nervous system. Annu. Rev. Cell Dev. Biol. 28, 555–573. 10.1146/annurev-cellbio-101011-155822, PMID: 23057747

[ref175] Thomson T. Lin H. (2009). The biogenesis and function of PIWI proteins and piRNAs: progress and prospect. Annu. Rev. Cell Dev. Biol. 25, 355–376. 10.1146/annurev.cellbio.24.110707.175327, PMID: 19575643 PMC2780330

[ref176] Treiber C. D. Waddell S. (2017). Resolving the prevalence of somatic transposition in Drosophila. elife 6, pii: e28297. 10.7554/eLife.28297, PMID: 28742021 PMC5553932

[ref177] Tritto P. Specchia V. Fanti L. Berloco M. D’Alessandro R. Pimpinelli S.. (2003). Structure, regulation and evolution of the crystal-Stellate system of Drosophila. Genetica 117, 247–257. 10.1023/A:1022960632306, PMID: 12723704

[ref178] Tucker B. Richards R. Lardelli M. (2004). Expression of three zebrafish orthologs of human FMR1-related genes and their phylogenetic relationships. Dev. Genes Evol. 214, 567–574. 10.1007/s00427-004-0438-9, PMID: 15378363

[ref179] Upton K. R. Gerhardt D. J. Jesuadian J. S. Richardson S. R. Sanchez-Luque F. J. Bodea G. O.. (2015). Ubiquitous L1 mosaicism in hippocampal neurons. Cell 161, 228–239. 10.1016/j.cell.2015.03.026, PMID: 25860606 PMC4398972

[ref180] Vagin V. V. Sigova A. Li C. Seitz H. Gvozdev V. Zamore P. D. (2006). A distinct small RNA pathway silences selfish genetic elements in the germline. Science 313, 320–324. 10.1126/science.1129333, PMID: 16809489

[ref181] Verrotti A. C. Wharton R. P. (2000). Nanos interacts with cup in the female germline of Drosophila. Development 127, 5225–5232. PMID: 11060247 10.1242/dev.127.23.5225

[ref182] Wan L. Dockendorff T. C. Jongens T. A. Dreyfuss G. (2000). Characterization of dFMR1, a Drosophila melanogaster homolog of the fragile X mental retardation protein. Mol. Cell. Biol. 20, 8536–8547. 10.1128/MCB.20.22.8536-8547.2000, PMID: 11046149 PMC102159

[ref183] Watanabe T. Takeda A. Tsukiyama T. Mise K. Okuno T. Sasaki H.. (2006). Identification and characterization of two novel classes of small RNAs inthe mouse germline: retrotransposon-derived siRNAs in oocytes and germline small RNAs in testes. Genes Dev. 20, 1732–1743. 10.1101/gad.1425706, PMID: 16766679 PMC1522070

[ref184] Weick E. M. Miska E. A. (2014). piRNAs: from biogenesis to function. Development 141, 3458–3471. 10.1242/dev.094037, PMID: 25183868

[ref185] Weisz E. D. Monyak R. E. Jongens T. A. (2015). Deciphering discord: how Drosophila research has enhanced our understanding of the importance of FMRP in different spatial and temporal contexts. Exp. Neurol. 274, 14–24. 10.1016/j.expneurol.2015.05.015, PMID: 26026973 PMC12047081

[ref186] West A. E. Greenberg M. E. (2011). Neuronal activity-regulated gene transcription in synapse development and cognitive function. Cold Spring Harb. Perspect. Biol. 3, pii: a005744. 10.1101/cshperspect.a005744, PMID: 21555405 PMC3098681

[ref187] Xiol J. Spinelli P. Laussmann M. A. Homolka D. Yang Z. Cora E.. (2014). RNA clamping by Vasa assembles a piRNA amplifier complex on transposon transcripts. Cell 157, 1698–1711. 10.1016/j.cell.2014.05.018, PMID: 24910301

[ref188] Xu X. L. Li Y. Wang F. Gao F. B. (2008). The steady-state level of the nervous-system-specific microRNA-124a is regulated by dFMR1 in Drosophila. J. Neurosci. 28, 11883–11889. 10.1523/JNEUROSCI.4114-08.2008, PMID: 19005053 PMC2605156

[ref189] Yang L. Duan R. Chen D. Wang J. Jin P. (2007). Fragile X mental retardation protein modulates the fate of germline stem cells in Drosophila. Hum. Mol. Genet. 16, 1814–1820. 10.1093/hmg/ddm129, PMID: 17519221

[ref190] Yang Y. Xu S. Xia L. Wang J. Wen S. Jin P.. (2009). The bantam microRNA is associated with drosophila fragile X mental retardation protein and regulates the fate of germline stem cells. PLoS Genet. 5:e1000444. 10.1371/journal.pgen.1000444, PMID: 19343200 PMC2654963

[ref191] Yu Z. Fan D. Gui B. Shi L. Xuan C. Shan L.. (2012). Neurodegeneration-associated TDP-43 interacts with fragile X mental retardation protein (FMRP)/Staufen (STAU1) and regulates SIRT1 expression in neuronal cells. J. Biol. Chem. 287, 22560–22572. 10.1074/jbc.M112.357582, PMID: 22584570 PMC3391095

[ref192] Zalfa F. Eleuteri B. Dickson K. S. Mercaldo V. De Rubeis S. di Penta A.. (2007). A new function for the fragile X mental retardation protein in regulation of PSD-95 mRNA stability. Nat. Neurosci. 10, 578–587. 10.1038/nn1893, PMID: 17417632 PMC2804293

[ref193] Zhang M. Wang Q. Huang Y. (2007). Fragile X mental retardation protein FMRP and the RNA export factor NXF2 associate with and destabilize Nxf1 mRNA in neuronal cells. Proc. Natl. Acad. Sci. U. S. A. 104, 10057–10062. 10.1073/pnas.0700169104, PMID: 17548835 PMC1891223

[ref194] Zhang W. Cheng Y. Li Y. Chen Z. Jin P. Chen D. (2014). A feed-forward mechanism involving Drosophila fragile X mental retardation protein triggers a replication stress-induced DNA damage response. Hum. Mol. Genet. 23, 5188–5196. 10.1093/hmg/ddu241, PMID: 24833720 PMC4159158

[ref195] Zhang Y. Brown M. R. Hyland C. Chen Y. Kronengold J. Fleming M. R.. (2012). Regulation of neuronal excitability by interaction of fragile X mental retardation protein with slack potassium channels. J. Neurosci. 32, 15318–15327. 10.1523/JNEUROSCI.2162-12.2012, PMID: 23115170 PMC3518385

[ref196] Zhang Y. O’Connor J. P. Siomi M. C. Srinivasan S. Dutra A. Nussbaum R. L.. (1995). The fragile X mental retardation syndrome protein interacts with novel homologs FXR1 and FXR2. EMBO J. 14, 5358–5366. 10.1002/j.1460-2075.1995.tb00220.x, PMID: 7489725 PMC394645

[ref197] Zhang Y. Q. Bailey A. M. Matthies H. J. Renden R. B. Smith M. A. Speese S. D.. (2001). Drosophila fragile X-related gene regulates the MAP1B homolog Futsch to control synaptic structure and function. Cell 107, 591–603. 10.1016/S0092-8674(01)00589-X, PMID: 11733059

[ref198] Zhang Y. Q. Matthies H. J. Mancuso J. Andrews H. K. Woodruff E. 3rd Friedman D.. (2004). The Drosophila fragile X-related gene regulates axoneme differentiation during spermatogenesis. Dev. Biol. 270, 290–307. 10.1016/j.ydbio.2004.02.010, PMID: 15183715

[ref199] Zhang Z. Xu J. Koppetsch B. S. Wang J. Tipping C. Ma S.. (2011). Heterotypic piRNA Ping-Pong requires qin, a protein with both E3 ligase and Tudor domains. Mol. Cell 44, 572–584. 10.1016/j.molcel.2011.10.011, PMID: 22099305 PMC3236501

[ref200] Zhou R. Hotta I. Denli A. M. Hong P. Perrimon N. Hannon G. J. (2008). Comparative analysis of argonaute-dependent small RNA pathways in Drosophila. Mol. Cell 32, 592–599. 10.1016/j.molcel.2008.10.018, PMID: 19026789 PMC2615197

